# Adjustment of the Arabidopsis circadian oscillator by sugar signalling dictates the regulation of starch metabolism

**DOI:** 10.1038/s41598-017-08325-y

**Published:** 2017-08-16

**Authors:** Motohide Seki, Takayuki Ohara, Timothy J. Hearn, Alexander Frank, Viviane C. H. da Silva, Camila Caldana, Alex A. R. Webb, Akiko Satake

**Affiliations:** 1 Department of Biology, Faculty of Science, Kyushu University, 744 Motooka, Nishi-ku, Fukuoka 819-0395 Japan; 2 Graduate School of Environmental Science, Hokkaido University, N10W5, Sapporo, 060-0810 Japan; 3 Department of Plant Sciences, University of Cambridge, Cambridge, CB2 3EA United Kingdom; 4 Brazilian Bioethanol Science and Technology Laboratory (CTBE), Rua Giuseppe Máximo Scolfaro 10.000 CEP 13083-100 Campinas, São Paulo, Brazil; 5 Max Planck Partner Group at Brazilian Bioethanol Science and Technology Laboratory, Campinas, SP Brazil

## Abstract

Arabidopsis plants store part of the carbon fixed by photosynthesis as starch to sustain growth at night. Two competing hypotheses have been proposed to explain this diel starch turnover based on either the measurement of starch abundance with respect to circadian time, or the sensing of sugars to feedback to the circadian oscillator to dynamically adjust the timing of starch turnover. We report a phase oscillator model that permitted derivation of the ideal responses of the circadian regulation of starch breakdown to maintain sucrose homeostasis. Testing the model predictions using a sugar-unresponsive mutant of Arabidopsis demonstrated that the dynamics of starch turnover arise from the circadian clock measuring and responding to the rate of change of cellular sucrose. Our theory and experiments suggest that starch turnover is controlled by the circadian clock acting as a dynamic homeostat responding to sucrose signals to maintain carbon homeostasis.

## Introduction

The Earth’s rotation and revolution with the tilt of its axis create daily light and dark cycles and yearly seasonal changes in the timing of dawn and dusk. Most organisms on the Earth possess circadian clocks that coordinate metabolism and physiology with periodic environmental changes. Synchronizing circadian rhythms to environmental cues occurs through phase adjustments of the circadian clock, in the process of entrainment, which optimizes the circadian regulation of diverse biological processes, such as growth, stress responses, and reproduction^1^ and can result in an increased competitive advantage^2–4^.

In Arabidopsis, an important output of the circadian system is the timing of the diel turnover of starch^5, 6^. Arabidopsis stores a portion of the carbon fixed through sugars as insoluble starch, which accumulates during the day. At night, starch is broken down to provide sugars to sustain growth under dark. This process has several remarkable properties that are not easily explained within the context of known cellular signalling pathways. Starch abundance increases and decreases in an almost linear manner despite the exponential dynamics of cellular processes. Starch is consumed during night and reaches a minimum almost precisely at dawn regardless of changing photoperiod, due to regulation from the circadian oscillator^7, 8^. The system adjusts the rate of change of starch accumulation and loss in response to seasonal changes of photoperiod, such that Arabidopsis plants in shorter photoperiods accumulate starch faster during the day and lose starch more slowly at night than those grown in longer photoperiods^7–11^. Lastly, early, or late onset of night, causes an immediate change in the rate of loss of starch abundance^7, 8^.

The molecular mechanisms that coordinate the turnover of starch with the external photoperiod remain unknown. Mathematical modelling aimed at providing a theoretical framework to consider the processes that regulate the diel turnover of starch has generated two competing hypotheses^12, 13^. In the first, it is assumed that the abundance of starch is measured^12, 14, 15^, whereas in the other, it is assumed that sucrose is measured^13, 16–18^. Both hypotheses consider that the circadian oscillator is required for the correct regulation of diel starch dynamics but with major differences in the assumed role of the clock. In the starch sensing models, the circadian clock is a passive timer used to measure the time of day^12, 14, 15^. In the second, sucrose feedbacks to the circadian clock to dynamically regulate the phase of the circadian oscillator^13, 16, 17^. There is strong experimental evidence for the role of the circadian oscillator in the timing of turnover of starch, which is critical to both hypotheses^11, 19^. In addition, inhibition of starch degradation rate in response to elevated sucrose levels, mediated by trehalose-6-phosphate (Tre6P) supports the hypothesis that sugar-sensing contributes to the regulation of starch turnover^20^. However, because of the interrelationships between starch, sugars and the clock it has been difficult using experimental data alone to differentiate between the profoundly different assumptions of starch or sucrose sensing and the role of dynamical feedback from sucrose to the circadian clock.

Our recent experimental finding that sugars can adjust the phase and period of the circadian oscillator of Arabidopsis^21^ motivated us to examine further the hypothesis that the diel turnover of starch arises from sucrose-sensing and feedback to the circadian oscillator^16, 17^. We tested this hypothesis using both modelling and experimental approaches. Because previous models considering the feedback of sucrose status to the circadian oscillator^16, 17^ did not explicitly formalize the phase-dependent response of the circadian clock to sucrose, we developed a new phase oscillator model, which describes the adjustment of the circadian oscillator as a function of the phase at which the sucrose signal is received. The phase shift of the circadian clock by metabolism has also been reported in feeding and fasting cycles in mammals^22^ and rhythmic feeding cycles in cyanobacteria^23^, suggesting the role of metabolism as an important synchronizer of the circadian clock in diverse organisms. Using our model, we demonstrated how the circadian oscillator in Arabidopsis shows a phase advance in the subjective morning and a phase delay at night in response to sugar signals^21^. We then predicted that the metabolic regulation of the circadian clock contributes to appropriate carbon use in changing photoperiods. Predictions arising from the phase oscillator model were tested in a mutant in which the circadian oscillator does not respond to sugars. In wild type plants, sugars advance the phase of the circadian oscillator in the morning, but this response is absent in plants in which the circadian oscillator gene *PSEUDO RESPONSE REGULATOR 7* is not functional (*prr7-*11)^21^. Using *prr7-11* we demonstrate that dynamic sensing of sucrose by the circadian oscillator is required for the correct regulation of the diel turnover of starch in response to altered photoperiod.

## Results

### Linear starch dynamics is an emergent property that arises from sucrose homeostasis

**Figure 1:**
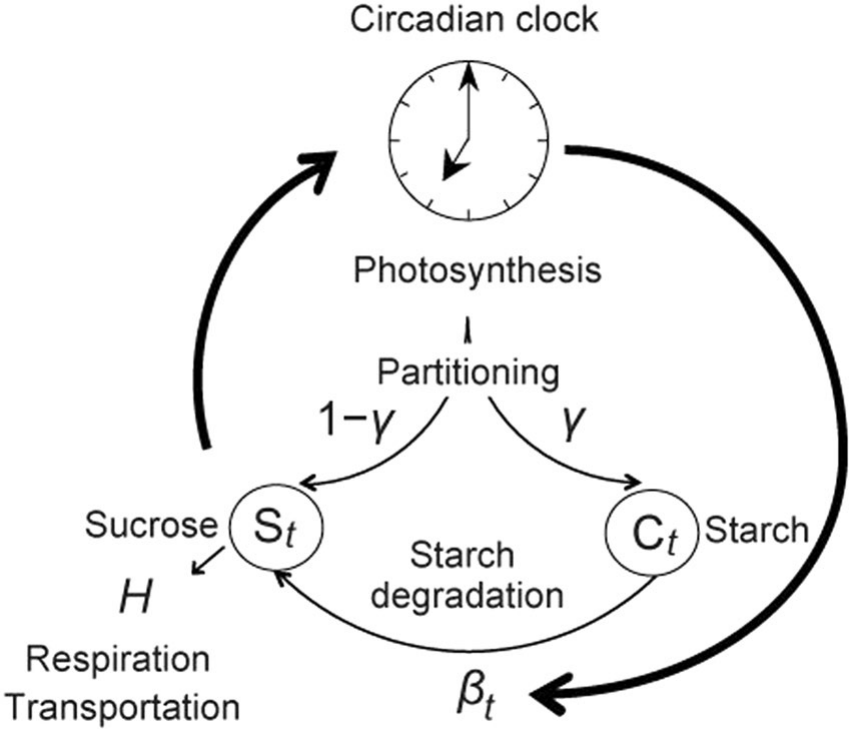
The model describing the feedback between the circadian clock and carbon metabolism. The circadian clock regulates the starch degradation process and thus influences the diel sucrose profile. Furthermore, feedback from the sucrose status can adjust the phase of circadian clock.

To theoretically assess the diel turnover of starch in the light of empirical data demonstrating a feedback from sugar sensing to the circadian system^21^, we combined a phase oscillator model of the dynamical adjustment of the circadian system with a description of carbon metabolism. We first explain the model of carbon metabolism, which is similar to that we have used previously^16^ and perform extended analysis to demonstrate that linear starch dynamics can arise from the homeostatic regulation of sucrose in the cell^17^. In the model, the following assumptions about the dynamics for sucrose (*S*_*t*_) and starch (*C*_*t*_) are made. During the light period photoassimilates are produced at a constant rate^4, 11^ *a*. A fraction, *γ*, of those photoassimilates is partitioned into starch for storage, and the remaining portion, 1 − *γ*, is transformed into sucrose for use in respiration or transportation at rate *H* (Fig. 1). The partitioning rate into starch (*γ*) is likely to be regulated by the balance between photoassimilated carbon and the rate of sucrose synthesis, which is mediated by the exchange of triose phosphate generated in the chloroplast for inorganic phosphate generated in the cytosol^19, 24–26^, but this regulation solely cannot explain the control of carbon availability over the diel cycle^27–29^. To produce sucrose, starch is degraded at a rate of *β*_*t*_ per unit surface area of the starch granule. Here, we call *β*_*t*_ the “starch degradation rate.” In an original model^16, 17^, we considered that all the rates for carbon partitioning (*γ*), sucrose transportation (*H*), and starch degradation (*β*_*t*_) oscillate under the influence of the circadian clock. Here we simplified the model by considering the constant rates for *γ* and *H* because oscillations of *γ* and *H* were less important to explain observed starch dynamics^17^. We normalized the length of a day as 1, and the fractions of light and dark periods in a day are given as *τ*_L_ and *τ*_D_, respectively (*τ*_L_ + *τ*_D_ = 1). Based on these assumptions, the dynamics of sucrose and starch are represented with the following equations:

1 $${\dot{S}}_{t}=\{\begin{array}{cc}a(1-\gamma )+{\beta }_{t}{{C}_{t}}^{\kappa }-H{S}_{t} & {\rm{u}}{\rm{n}}{\rm{d}}{\rm{e}}{\rm{r}}\,{\rm{l}}{\rm{i}}{\rm{g}}{\rm{h}}{\rm{t}}\\ {\beta }_{t}{{C}_{t}}^{\kappa }-H{S}_{t} & {\rm{u}}{\rm{n}}{\rm{d}}{\rm{e}}{\rm{r}}\,{\rm{d}}{\rm{a}}{\rm{r}}{\rm{k}}\end{array},$$

2 $${\dot{C}}_{t}=\{\begin{array}{cc}a\gamma -{\beta }_{t}{{C}_{t}}^{\kappa } & {\rm{u}}{\rm{n}}{\rm{d}}{\rm{e}}{\rm{r}}\,{\rm{l}}{\rm{i}}{\rm{g}}{\rm{h}}{\rm{t}}\\ -{\beta }_{t}{{C}_{t}}^{\kappa } & {\rm{u}}{\rm{n}}{\rm{d}}{\rm{e}}{\rm{r}}\,{\rm{d}}{\rm{a}}{\rm{r}}{\rm{k}}\end{array}.$$

Starch-degrading enzymes cannot access all available starch because starch exists as large polymers (granules), and the degradation process only occurs at the surface of each granule. Thus, we assume that starch degradation occurs in proportion to the surface area of starch granule represented as *C*_*t*_^*κ*^ in Eqs (1) and (2). We mainly assumed *κ* = 2/3 (Supplementary Table S1), which is widely applied for three-dimensional objects, and confirmed that the value of *κ* has small effect on the model outcomes (Supplementary Fig. S1). Although a generic rate law for surface-active enzymes suggested the importance to consider the available surface area for enzymes^30^, the surface area of starch granules would not be a rate limiting factor for starch degradation^14^. The saturating degradation of starch can also be considered if Michaelis–Menten kinetics are incorporated into the mathematical model^31, 32^. However, previous studies showed that this type of kinetics failed to explain the instant change of starch loss rate in response to an unexpectedly early or late onset of night^14, 17^. By combining Eqs (1) and (2), we derived the following equation:

3 $${\dot{S}}_{t}=\{\begin{array}{cc}a-{\dot{C}}_{t}-H{S}_{t} & {\rm{u}}{\rm{n}}{\rm{d}}{\rm{e}}{\rm{r}}\,{\rm{l}}{\rm{i}}{\rm{g}}{\rm{h}}{\rm{t}}\\ -{\dot{C}}_{t}-H{S}_{t} & {\rm{u}}{\rm{n}}{\rm{d}}{\rm{e}}{\rm{r}}\,{\rm{d}}{\rm{a}}{\rm{r}}{\rm{k}}\end{array}.$$

Based on Eq. (3), we can generate the following equation:

4 $${\dot{S}}_{t}=0\,\Longleftrightarrow\, {\dot{C}}_{t}=\{\begin{array}{cc}a-H{S}_{t} & {\rm{u}}{\rm{n}}{\rm{d}}{\rm{e}}{\rm{r}}\,{\rm{l}}{\rm{i}}{\rm{g}}{\rm{h}}{\rm{t}}\\ -H{S}_{t} & {\rm{u}}{\rm{n}}{\rm{d}}{\rm{e}}{\rm{r}}\,{\rm{d}}{\rm{a}}{\rm{r}}{\rm{k}}\end{array}.$$

The left-hand side of Eq. (4) shows that the sucrose level does not change over time and, thus, is kept constant. Parameters *a* and *H* on the right-hand side of Eq. (4) are constant; therefore, if the sucrose level does not change, then the starch amount changes constantly with a linear increase during the day and linear decrease at night.

These results show that when the level of sucrose is constant, the starch profile is always linear and *vice versa*. Furthermore, this relationship suggests that the linearity of starch turnover is an emergent property of sucrose homeostasis. We consider that there to be a reserve pool that includes starch but might also include organic acids (such as malate and fumarate). Thus the same logic for sucrose homeostasis can be approximately applied to the total pool of soluble carbon, including glucose, fructose and organic acids. The emergent nature of the starch profile in our model represents a major difference from models in which starch sensing is assumed and specific chemical kinetics are assumed to realize the linear starch profiles^14^. Our finding that linear starch kinetics can arise as an emergent property of homeostasis of sugars is consistent with the empirical data that demonstrate that rather stable sucrose levels are found through the day although starch levels change drastically through the diel cycle^11, 28^. The idealized relationship between starch and sucrose presented here is realized even under fluctuating environments due to a feedback mechanism from sucrose to the circadian clock to minimize sucrose changes, as we explain later.

### The role of phase adjustment of the circadian oscillator in starch metabolism

**Figure 2:**
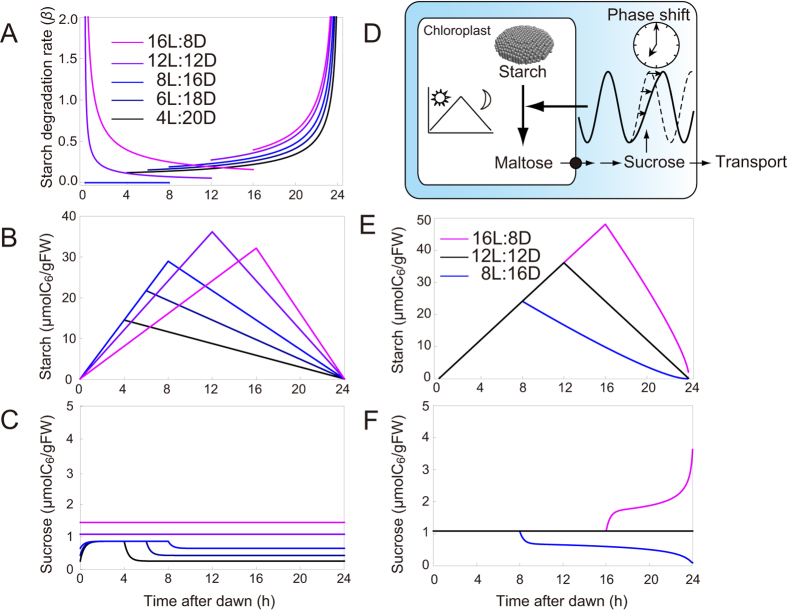
(**A**) Ideal profiles for the diel starch degradation rate (*β*). (**B**) Starch and (**C**) sucrose profiles for different light and dark cycles. Results from different light and dark cycles were plotted with different colours. (**D**) An illustration of phase shift of the circadian clock in response to changing sucrose levels. When the plant cannot change phase of its circadian clock by sugars, the arrow from sucrose to the clock disappears. (**E**) Starch and (**F**) sucrose profiles when a plant with a starch degradation rate optimized for 12 L:12D conditions (black) is transferred to a long (16 L:8D; purple) or short (8 L:16D; blue) photoperiod. Parameter values used for the analyses are listed in Supplementary Table S1.

We previously demonstrated that the nearly linear dynamic of starch accumulation and loss is caused by a non-linear starch degradation rate. We determined the starch degradation rate (*β*_*t*_) required to achieve sucrose homeostasis in a given photoperiod (Supplementary Methods)^13, 17^, and showed that it has a peak at dawn regardless of the magnitude of *κ* in Eqs (1) and (2) (Fig. 2A), and it is independent from the amount of starch at dusk [Eqs (S17) and (S22)]. This result is clearly different from the previous models assuming that the plants sense the starch amount at dusk and regulates starch degradation accordingly^14, 15^. We assume that this diel fluctuation of starch degradation is generated under the influence of circadian clock because there is an increasing evidence that the activity of rate-limiting enzymes shows clear diel oscillation due to the regulation by the circadian clock in animals^33, 34^, although detailed mechanisms underlying oscillating activity of starch degradation enzymes still remain unknown in plants. *β*_*t*_ is positive in the light when photoperiod is long (Fig. 2A), suggesting that starch degradation could occur in the light. Recent experimental studies have supported our theoretical finding that the starch degradation occurs during the light^35, 36^, although the mechanism remains not elucidated^37^. The discontinuous feature of *β*_*t*_ at dusk (Fig. 2A) implies that the starch degradation rate is regulated in a day mode and a night mode, possibly as a consequence of dual regulation by light signalling in addition to the circadian oscillator^38^.

The theoretically determined starch degradation rate captures an important feature of the data: the starch accumulation rate increased, and the rate of the loss of starch decreased as the photoperiod shortened (Fig. 2B), while the sucrose level is less variable even under different photoperiod conditions (Fig. 2C). In short photoperiods (4, 6, and 8 h) there was no difference in the rate of starch accumulation rate as observed empirically (Fig. 2B)^28^ demonstrating that the model can simulate the observed starch dynamics without those dynamics being explicitly assumed. We also demonstrate that the stationary levels of sucrose under light and dark conditions cannot be the same when the fraction of light period is smaller than the carbon partitioning rate (Fig. 2C; Supplementary Methods).

The timing of the optimal starch degradation rate to achieve homeostasis changes with photoperiod, which probably involves the circadian oscillator^8, 14, 16^. To illustrate the importance of the adjustment of starch degradation rates with changing photoperiods, we considered the implication on starch dynamics if the plant could not change phase of its circadian clock by light and/or sugars (Fig. 2D). We did this by using the endogenous oscillation of the starch degradation rate that was optimized to maintain sucrose homeostasis for a 12 h photoperiod. In a 12 h photoperiod the model achieved sucrose homeostasis with a linear diel starch profile (Fig. 2E,F). When the external photoperiod was lengthened beyond 12 h, the rate of nocturnal starch loss increased and when the photoperiod was shortened to 8 h the night time starch loss rate decreased (Fig. 2E). These simulations suggest that even in the absence of phase adjustment of the circadian clock, the plant can appropriately respond to an unexpected early or late dusk^7, 8, 14^ because the night-time starch degradation rate required for sucrose homeostasis is almost invariable regardless of photoperiod (Fig. 2A). However, in the absence of phase adjustment of the circadian oscillator the plant in the model cannot alter its starch accumulation rate during the day (Fig. 2E), which contradicts the empirical findings that starch accumulation rates change flexibly in response to the photoperiod^7, 8, 11^. The failure to adjust the starch accumulation rate in the day is accompanied by a loss of sucrose homeostasis (Fig. 2F). The theoretical results suggest that in changing photoperiods circadian adjustment is required to readjust the starch degradation rate to regulate starch accumulation during the day and maintain sucrose homeostasis.

### Optimal phase shifts for sustaining sucrose homeostasis

Because we found above that adjustment of circadian phase in the light is required for sucrose homeostasis, we extended our model by incorporating the phase-dependent responses of the circadian clock to light and sugar. Although detailed entrainment mechanisms of the clock are unknown, entrainment is thought to be accomplished by resetting events that correct for the deviations of the endogenous cycle from the external one^39^. The most reliable indicators of solar day length are dawn and dusk, and most organisms have evolved to use these transitions as their primary zeitgeber for circadian phase adjustments^40^. In addition, sugar adjusts the phase of Arabidopsis circadian oscillator by phase advance in the subjective morning^21^.

In an extended model, the starch degradation rate is given as the function of the phase (*φ*) of the circadian oscillator, which can be formalized by the following phase oscillator dynamics:

5 $${\dot{\phi }}_{t}=\omega +{Z}_{{\rm{L}}}({\phi }_{t}){f}_{{\rm{L}}}({\mathop{L}\limits^{ \sim }}_{t})+{Z}_{{\rm{S}}}({\phi }_{t}){f}_{{\rm{S}}}({\mathop{S}\limits^{ \sim }}_{t}),$$

where *ω* is the angular frequency, and *Z*_L_(*φ*_*t*_) and *Z*_S_(*φ*_*t*_) are the phase-dependent sensitivity functions for light and sugar signals, respectively. ${f}_{{\rm{L}}}({\tilde{L}}_{t})$ and ${f}_{{\rm{S}}}({\tilde{S}}_{t})$ indicate input from light and sucrose signals denoted as ${\tilde{L}}_{t}$ and ${\tilde{S}}_{t}$, respectively (see Supplementary Methods for more details). We assumed that light pulses at dawn and dusk set the phase of the oscillator so that it equals the external time (Supplementary Methods). The period of the oscillator was set to be 24 h, although slight deviations from the 24 h period do not affect our results. The starch degradation rate *β*_*t*_ in Eqs (1) and (2) is replaced as a function of the phase of circadian clock: $\tilde{\beta }({\phi }_{t})$ (Supplementary Methods).

**Figure 3:**
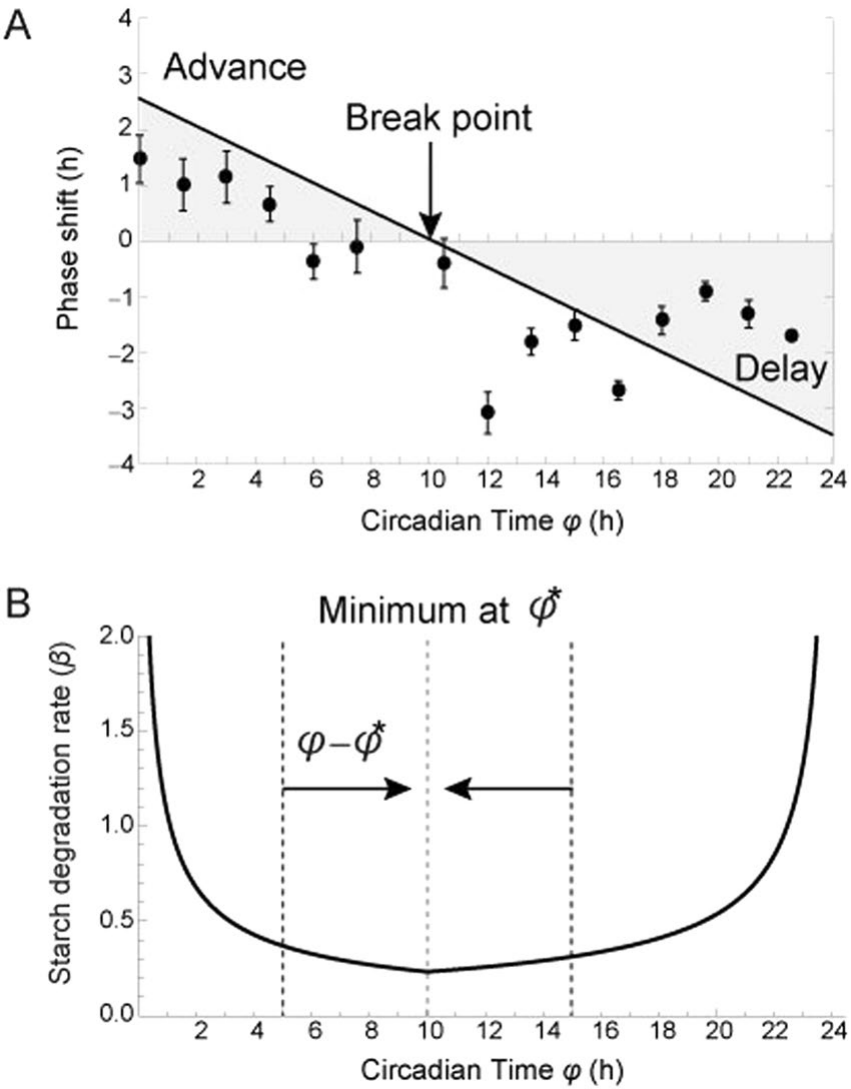
(**A**) Comparison between the experimentally derived phase response curve (PRC) and the theoretically derived PRC. Dots represent the Col-0 *CCA1:LUC* phase response curve to pulses of 90 mM sucrose in constant 10 µmol m^−2^ s^−1^ red/blue light. The phase difference in the oscillation of *CCA1:LUC* for the circadian cycle following sucrose pulse is plotted. Data represent the mean ± SEM (*n* = 8). The phase response is normalized to a 24 h period. A line represents the theoretically derived PRC when the minimum starch degradation rate occurs at a circadian time of 10 h after subjective dawn and the strength of sugar input signal [${f}_{{\rm{S}}}({\tilde{S}}_{t})$ in Eq. (5)] is 0.25. The experimental data are available online (Supplementary Dataset 1). (**B**) Optimal phase shift for sucrose homeostasis. φ* represents the subjective time at which the starch degradation rate is at its minimum. *φ* is the circadian time when the sugar pulse was added.

The product, *Z*_S_(*φ*_*t*_)${f}_{{\rm{S}}}({\tilde{S}}_{t})$, in Eq. (5) corresponds to the general phase response curve (PRC) for the pulse of sucrose signals given at the phase *φ*_*t*_. The magnitude of phase shift to a sucrose signal at a certain circadian time was determined empirically by a sugar pulse experiment using plants in which the endogenous sugars are depleted but not sufficiently to cause growth cessation (Fig. 3A)^21^. To explain why the empirically measured PRC had a phase advance in the morning and phase delay in the evening and night (Fig. 3A)^21^, we theoretically derived the PRC needed to minimize fluctuation of sucrose levels. We used a simulated experiment in which the maximum level of sucrose signal is received by the plant at a certain circadian time *φ*_*t*_ = *φ*. In order to minimize sucrose fluctuations, the plant would transiently reduce the sucrose production by downregulating starch degrading activity. We assume that downregulation of starch degrading activity is derived from the phase shift of the circadian clock. When the starch degradation rate $\tilde{\beta }({\phi }_{t})$ has a minimum at *φ*_*t*_ = φ* (Fig. 3B), the phase shift from *φ*_*t*_ = *φ* to *φ*_*t*_ = φ* is the best solution for minimizing the sucrose fluctuation because decreasing the starch degradation rate is the most effective way to mitigate a transient elevation in sugar levels (Fig. 3B). Thus, the optimal phase shift to the sugar pulse given at *φ*_*t*_ = *φ* is φ* − *φ*, which is positive (phase advance) until *φ*_*t*_ = φ*, zero at *φ*_*t*_ = φ* (the break point), and negative (phase delay) after *φ*_*t*_ = φ* (Fig. 3A). Therefore, the assumption that the plant minimizes sucrose fluctuations naturally derives the phase advance in the subjective morning and delay at night, which is qualitatively the same as the observed phase shifts (Fig. 3A)^21^. The position of the break point in the optimal PRC is determined by the circadian time when the starch degradation rate is at its minimum. The experimentally-derived PRC (Fig. 3A) suggests that the minimum starch degradation rate is attained approximately 6–11 h after the subjective dawn. The optimal PRC for sucrose homeostasis is also confirmed by numerical calculation that determines the phase responses minimizing the deviation from sucrose homeostasis (Supplementary Methods; Supplementary Fig. S2). Even if different functions for starch degradation rate are applied, the same PRC is obtained for optimal sucrose homeostasis as long as the starch degradation rate has a single minimum at the same circadian time.

### Phase adjustment of the circadian oscillator by sucrose is necessary to achieve sucrose homeostasis

Next, we investigated whether the optimal phase response of the circadian oscillator to a sugar signal is effective at adjusting the rate of starch accumulation in response to changes in photoperiod. We applied the optimal PRC with the break point at 10 h after subjective dawn to the phase oscillator dynamics in Eq. (5) by assuming that the subjective starch degradation rate is optimized for a 10 h photoperiod. Other functions for the starch degradation rate can be used as long as the starch degradation rate has a single minimum at the same circadian time. We also assumed that plants sense the rate of change in sugar levels as a signal (i.e., ${\tilde{S}}_{t}={\dot{S}}_{t}$, where ${\dot{S}}_{t}$ indicates the time derivative of the sucrose level). When ${\dot{S}}_{t}$ is negative (i.e., sucrose level decreases), an opposite phase shift occurs when compared with a positive ${\dot{S}}_{t}$ (i.e., sucrose level increases). Since we found that adjustment of phase affects only the diel turnover of starch during the day, and not during night (Fig. 2E), we consider first only the effect of phase adjustment of the circadian oscillator by sugars during the light period.

**Figure 4:**
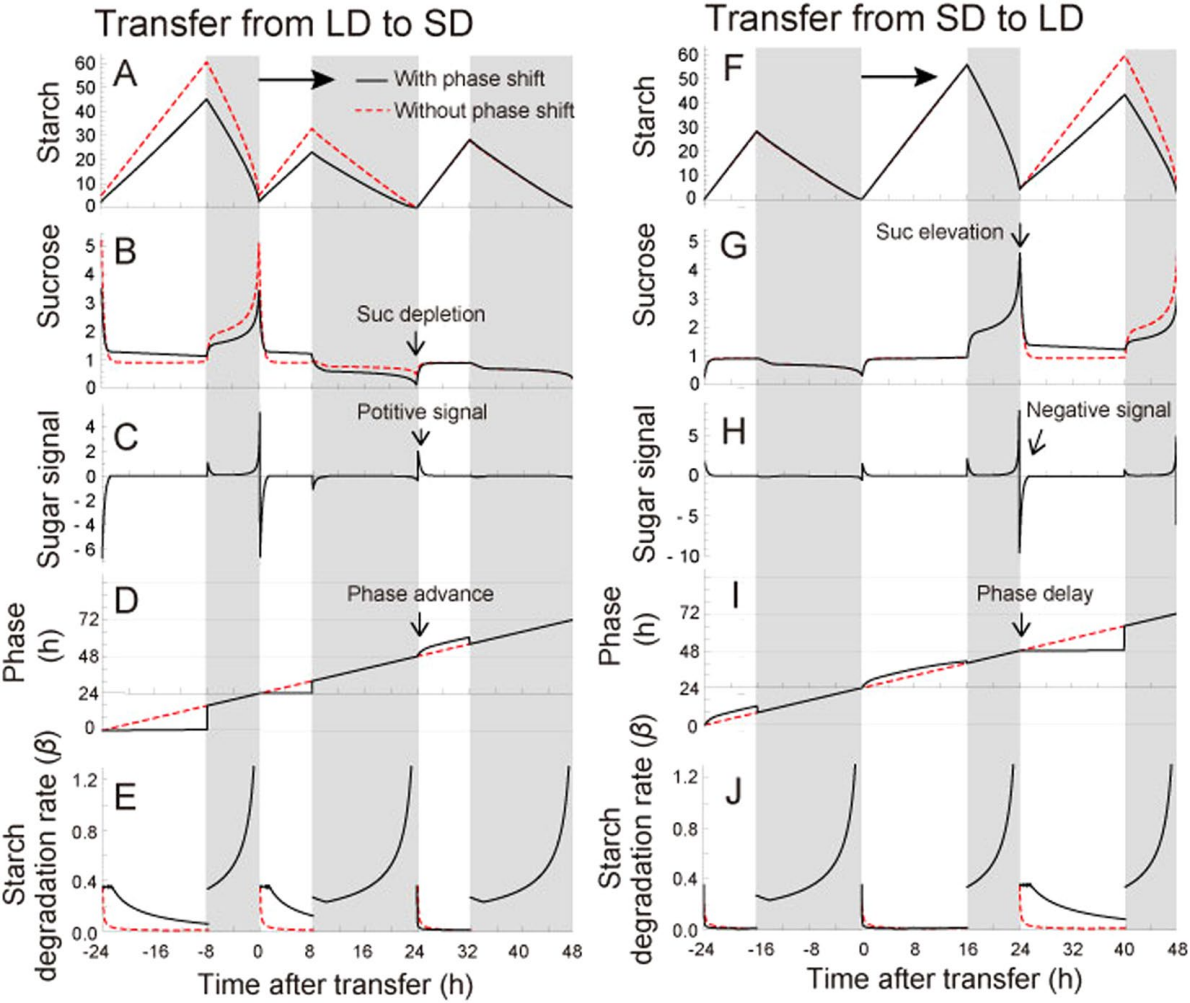
(**A**) Predicted starch profile, (**B**) sucrose profile, (**C**) sugar signal, (**D**) phase change, and (**E**) starch degradation rate of the plant that was transferred from long (16 L:8D) to short (8 L:16D) days. (**F**) Predicted starch profile, (**G**) sucrose profile, (**H**) sugar signal, (**I**) phase change, and (**J**) starch degradation rate of the plant that was transferred from short (8 L:16D) to long (16 L:8D) days. Black lines represent the plant with a phase shift, and red lines represent the plant without a phase shift. The break point of the PRC was set at 10 h. Parameter values used for the analyses are listed in Supplementary Table S1. The unit for starch and sucrose is µmol C_6_ g^−1^ FW. Parameter values used for the Hill function in Eq. (S29) are (*K*, *n*) = (0.1, 1.0).

In the numerical analyses of the model, the rate of starch loss at night decreased (Fig. 4A), and the amount of sucrose at the end of the day decreased on the first day (Fig. 4B) when plants were transferred from long day [LD; 16 h light (L): 8 h dark (D)] to short day (SD; 8 L:16D). This generated a positive sugar signal in the morning because the sugar level was elevated rapidly after dawn (Fig. 4C), which advanced the phase of the circadian oscillator (Fig. 4D) and the starch degradation rate was decreased on the second day after transfer (Fig. 4E). This result suggests that the phase adjustment of the circadian oscillator by sugar effectively buffers the decrease in sucrose at the end of the day by increasing the starch accumulation rate on the second day after transfer (Fig. 4A,B). The increased starch accumulation rate did not occur when circadian phase adjustment by sugar was not incorporated (Fig. 4A).

In contrast, when plants were transferred from SD to LD conditions, the sucrose level was elevated by the end of the day, which generated a negative sugar signal in the morning (Fig. 4H) because the sucrose level was decreased via a low starch degradation rate after dawn. The negative sugar signal delayed the phase of circadian oscillator (Fig. 4I), and the starch degradation rate increased on the second day after the transfer (Fig. 4J). Delayed phase of the core clock genes in longer photoperiods have been reported previously in experimental conditions^41^.

The increase in starch degradation rate due to phase adjustment of the circadian oscillator decreased the starch accumulation rate during the day and effectively buffered the increased level of sucrose at the end of the night (Fig. 4F,G). Again, the decreased starch accumulation rate did not occur when there was no phase adjustment by sugar. These results predict an essential role for the dynamic adjustment of the circadian clock by sugar to modulate starch metabolism in changing photoperiods. The role of phase shift in response to sugar signals was also stressed in the regulation of starch metabolism under fluctuating weather conditions (Supplementary Methods; Supplementary Fig. S3). The predicted starch profile (decreased starch loss rate at night under lowered light intensity) resembled the observed starch dynamics in the experiment controlling irradiance (Fig. 1 of ref. 42).

Changing the break point of the optimal PRC from 10 to 8 h after subjective dawn had little effect on the main results (Supplementary Fig. S4), suggesting that the position of break point for PRC is flexible. In contrast, sucrose homeostasis was not improved by sugar-mediated circadian phase adjustment when we assumed that the plants sense the sucrose concentration (i.e., ${\tilde{S}}_{t}={S}_{t}$) rather than the rate of change in sucrose levels (${\tilde{S}}_{t}={\dot{S}}_{t}$; Supplementary Fig. S5). This implies that plants sense sugar flux rather than direct sugar concentrations.

### Physiological experiments

We validated our model by comparing the predicted starch dynamics with experimental data obtained for Arabidopsis wild-type (Col-0) and a mutant in which the circadian clock does not respond to sugar signals (*pseudoresponse regulator 7–11*; *prr7-11*)^21^. The *prr7-11* mutant provides the critical test for the model because there is no effect on the circadian period of *CCA1:LUC* luminescence under the standard circadian testing conditions of constant light (80 μmol m^−2^ s^−1^) when compared with wild-type (Col-0: Mean ± SEM = 24.3 ± 0.1 h, *n* = 4; *prr7-11*: 24.7 ± 0.3 h, *n* = 4; *p* = 0.10; Supplementary Fig. S6). This feature distinguishes our investigation from a previous study in which circadian period was considered the defining variable and mutants were used that alter the free running period of the oscillator^8^. Here, we used a mutant that has wild type period under the experimental conditions but is compromised in the ability of the circadian oscillator to adjust phase in response to sugar signals to test the specific predictions that dynamic adjustment of the phase of the circadian system is required for the regulation of diel starch turnover^21^.

**Figure 5:**
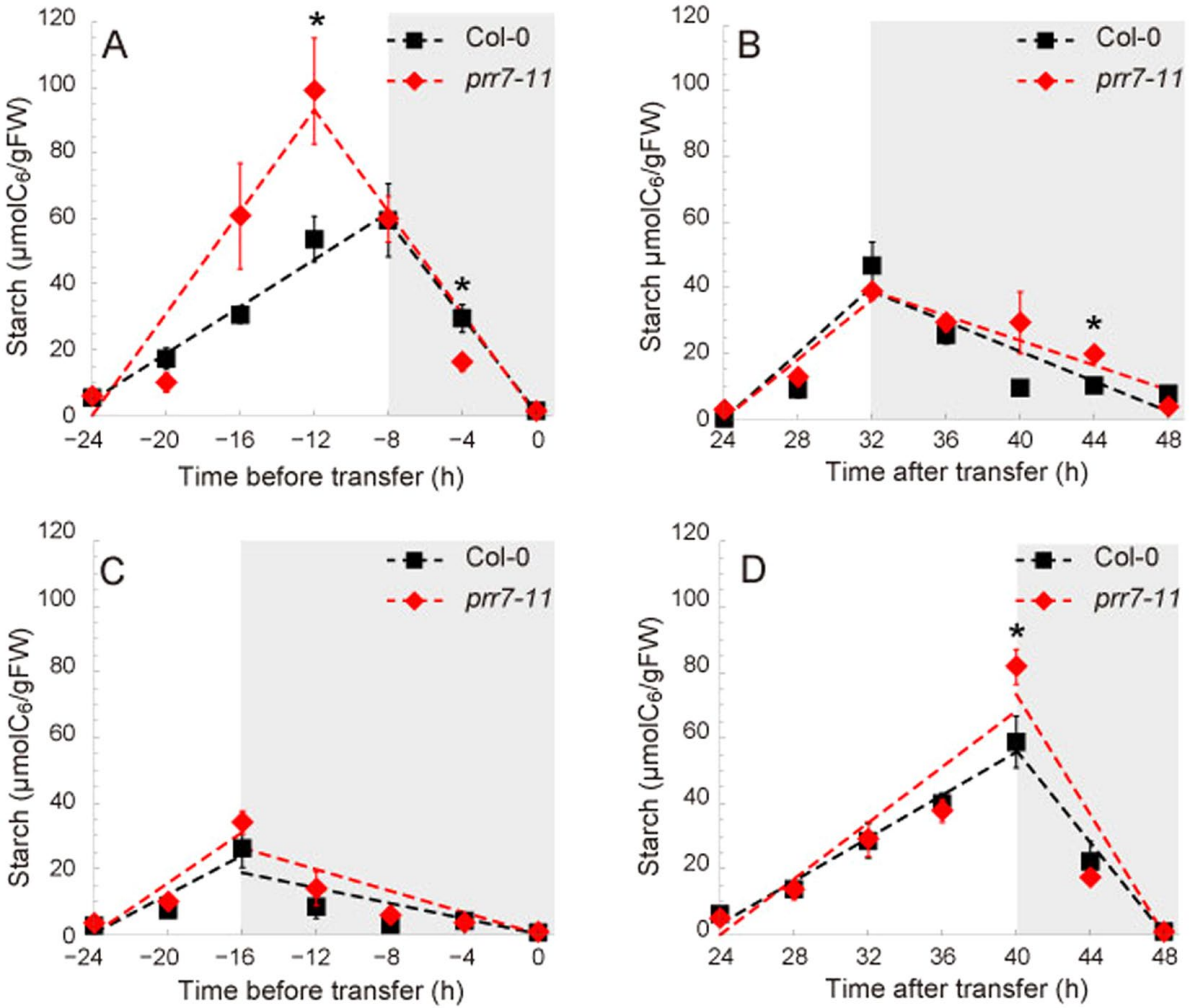
(**A**) Starch content in Col-0 and *prr7-11* plants grown in a 16 L:8D photoperiod and harvested from 28-day-old seedlings and (**B**) 30-day-old seedlings that were transferred to 8 L:16D after growing in standard LD conditions for 28 days. (**C**) The starch content in Col-0 and *prr7-11* grown in 8 L:16D photoperiod and harvested from 28-day-old seedlings and (**D**) 30-day-old seedlings that were transferred to 16 L:8D after growing in SD for 28 days. The light intensity was 100 µmol m^−2^ s^−1^. Asterisk (*) indicates *p* < 0.05 with Welch’s *t*-test, A–B *n* = 4–9, C–D *n* = 8. Dashed lines indicate linear regression lines for starch accumulation or loss (Supplementary Table S2). The experimental data are available online (Supplementary Datasets 2–3).

Our model predicted that phase adjustment by sugars would result in a decreased level of starch accumulated during the light period under specific LD conditions (16 L:8D), and a mutant lacking this response to sucrose would accumulate more starch than the wild-type (Fig. 4A). As predicted by the model, *prr7-11* had higher peak starch concentration when compared with Col-0. Starch concentration peaked prior to the end of the photoperiod in *prr7-11* at ZT12, with a statistically significant difference compared to Col-0 at ZT12 (Col-0: 53.66 ± 7.05 µmol C_6_ g^−1^ FW, *n* = 7; *prr7-11*: 98.90 ± 16.19 µmol C_6_ g^−1^ FW, *n* = 9; Welch’s *t*-test, *p* = 0.03; Fig. 5A). Under this LD condition, we also found that starch accumulation rate of *prr7-11* was significantly higher than that of Col-0 (linear regression, Col-0: 3.58 ± 0.47 µmol C_6_ g^−1^ FW h^−1^; *prr7-11*: 7.77 ± 0.82 µmol C_6_ g^−1^ FW h^−1^; analysis of covariance, *p* < 0.001; Fig. 5A; Supplementary Table S2). However, the mutant had a similar starch concentration to Col-0 when transferred to SD (8 L:16D) with no significant differences between concentrations at any time points during the day (Fig. 5B). Similarly, we tested the reciprocal model prediction concerning transfer from SD to LD (Fig. 4F). Again the model prediction was validated by the finding that *prr7-11* did not have significantly different starch concentrations compared to Col-0 when grown in SD (Fig. 5C), and when transferred to LD had significantly higher starch than Col-0 at ZT16 (Col-0: 58.75 ± 10.42 µmol C_6_ g^−1^ FW, *n* = 8; *prr7-11*: 81.68 ± 5.36 µmol C_6_ g^−1^ FW, *n* = 8; Welch’s *t*-test, *p* = 0.03; Fig. 5D). In addition, analyses of linear regression showed that *prr7-11* had a significantly higher starch accumulation rate during the light period (Col-0: 3.28 ± 0.36 µmol C_6_ g^−1^ FW h^−1^; *prr7-11*: 4.26 ± 0.23 µmol C_6_ g^−1^ FW h^−1^; analysis of covariance, *p* = 0.028; Fig. 5D; Supplementary Table S2) and starch loss rate during the dark period (Col-0: 7.03 ± 0.62 µmol C_6_ g^−1^ FW h^−1^; *prr7-11*: 9.16 ± 0.60 µmol C_6_ g^−1^ FW h^−1^; analysis of covariance, *p* = 0.018; Fig. 5D; Supplementary Table S2). We observed an early phase in peak starch concentration in *prr7-11* compared to Col-0 (Fig. 5A). When transferred to LD in *prr7-11* starch peaked at ZT16 (Fig. 5D), rather than ZT12 as seen for stable entrainment in LD (Fig. 5A). The different starch dynamics between *prr7-11* which were stably entrained to LD and those which were in the second cycle of transfer to LD might reflect the time it takes to adjust the circadian system to a new photoperiod due to after effects of light entrainment on the velocity of the circadian oscillator^43^. We observed a similar early phase in peak starch concentration in *prr7-11* in a 12 L:12D photoperiod, indicating that *prr7-11* also affects the timing of starch degradation (Supplementary Fig. S7), presumably through its effect on the phase of the circadian oscillator. Under this photoperiod regime starch concentration was higher in *prr7-11* (Col-0: 14.80 ± 3.34 µmol C_6_ g^−1^ FW, *n* = 6; *prr7-11*: 32.65 ± 3.71 µmol C_6_ g^−1^ FW, *n* = 6; Welch’s *t*-test, *p* = 0.0051; Supplementary Fig. S7) as predicted by the model (Supplementary Fig. S3). The results were confirmed by repeating the experiment independently (Supplementary Fig. S9; Supplementary Table S3).

Even when the feedback to the oscillator from sugar in both the day and night was considered in the model, it successfully predicted the change in starch accumulation and degradation rates during changing photoperiods (Supplementary Fig. S8). The distinctive result was that starch levels were elevated at the end of the night in LD conditions (Supplementary Fig. S8). This suggests that clock responses to sugar during the day alone are sufficient for the appropriate adjustment of starch turnover and the almost complete consumption of starch by the end of the night.

## Discussion

By formalizing the concepts derived from empirical studies into a phase oscillator model we have provided theoretical and experimental evidence that the circadian oscillator is regulated by sugar signals to achieve carbon homeostasis. The concept of homeostatic regulation of carbon can be approximately applied to the total pool of soluble carbon, including glucose, fructose and organic acids. Our results provide a theoretical background to explain why the experimentally-derived phase response to sucrose (Fig. 3)^21^ is advance in the morning and delay in the evening and night. Such a phase response to sugar signals was ideal to minimize sucrose fluctuations under changing environments. The incorporation of a theoretically-determined phase response to sucrose generated complex starch dynamics with non-intuitive predictions (Fig. 4). The model predicted the appropriate adjustment of starch turnover under changing photoperiods as a result of dynamic phase adjustment of the circadian oscillator and predicted the misregulation of starch dynamics in a sugar-insensitive mutant. This prediction was supported by experiments using the Arabidopsis *prr7-11* mutant, which has a circadian clock that does not adjust phase in response to sugars (Fig. 5)^21^. Moreover, the recent finding that the products of starch degradation are reduced by Tre6P^20, 44^ fits readily into the concept of sucrose homeostasis proposed in our model if it is assumed that Tre6P provides a measure of sugar status to the circadian clock.

We tried to distinguish between two competing hypotheses of the regulation of diel starch turnover^13–16^. If diel starch dynamics arise from a measurement of starch at dusk and circadian time, then the *prr7-11* mutant would be expected to have little effect, as the mutation has almost no significant effect on circadian period (Supplementary Fig. S6). On the other hand, if sucrose status is sensed and the circadian system adjusts dynamically to optimize carbon use, then *prr7-11* should affect starch turnover in a photoperiod-dependent manner. Indeed, *prr7-11* had increased starch abundance in LD conditions, which is in line with the model predictions (Fig. 5A,D). These results suggest that the timing mechanism that pairs the use of starch with the external photoperiod is likely to be based on sugar sensing^13, 16, 17^. However we cannot exclude the possibility that the differential starch profiles between *prr7-11* and wild type could be caused by other factors such as light signalling^45^ because the regulation of the circadian oscillator by light and sugar is tightly connected and might not be detected separately using current experimental techniques. Mathematical models provide a useful tool to decouple and separately assess the effects of light and sugar on the circadian clock and carbon metabolism.

It has been reported that when sugar sources are sufficient, starch was not completely degraded^42^. Moreover, change of temperature at night accelerated or slowed the starch loss rate even when the same amount of starch was accumulated at dusk (see Figs 4–6 of ref. 42). These data can be explained only by the model that takes into account the coupling of starch and sucrose dynamics.

Our results suggest that plants sense and respond to the rate of change in sucrose levels to regulate the circadian clock rather than responding to sucrose concentrations. This type of behaviour has been seen in *Bacillus subtillis* in which cells are responsive to the rate at which ethanol and salt stress increases^46^, as well as other bacterial systems which are sensitive to changes in their inputs, rather than to absolute levels^47, 48^. The rate responsive behaviour would function as a temporal filter that responds to rapidly growing signals more effectively than gradually developing one^46^. This temporal filter in sucrose signal would benefit plants to filter out noisy fluctuation in sucrose levels.

We conclude that the dynamic adjustment of the circadian system by sucrose contributes to carbon homeostasis and that the observed linear dynamics of starch turnover are an emergent behaviour arising from sucrose flux sensing. Entrainment of the circadian oscillator by light signals ensures that the circadian regulation of starch degradation occurs in synchronisation with the environment and adapts to changing day length. Adjustment of the phase of starch degradation by sugar signalling feedback to the circadian oscillator optimizes sucrose homeostasis (Fig. 2D), ensuring better carbon management for growth (and probably better entrainment to changing photoperiodic environments) than light entrainment alone^49^. Our model and data describe a new role for the circadian system in which the oscillator is more than a passive timer that synchronizes to environmental cues. The circadian system is a dynamic organizer with a plasticity of phase that contributes to carbon homeostasis and growth.

## Materials and Methods

### Plant material and growth conditions

Col-0 and *prr7-11* were gifts from Takeshi Mizuno^50^. The *prr7-11* allele was confirmed by genotyping upon receipt. Seeds were surface sterilized with 10% _w/v_ NaClO and 0.1% _w/v_ Triton X-100 (Fisher Scientific) and sown on half-strength Murashige and Skoog (Duchefa; 0.5 MS) 0.8% _w/v_ bactoagar (Becton–Dickinson) media adjusted to pH 5.7 with 0.1 M HCl (Fisher Scientific). Seeds were stratified for 72–96 h in the dark at 4 °C before being entrained in 12 L:12D, 16 L:8D, or 8 L:16D photoperiods at 19 °C in Sanyo growth cabinets supplied with 100–120 µmol m^−2^ s^−1^ of white fluorescent light.

### Starch measurements

Starch was determined in the insoluble material after ethanolic extraction of soluble sugars, followed by enzymatic digestion with α-amyloglucosidase and α-amylase^51^. Whole rosettes from three 21- to 28-day-old plants were used as a single sample. Samples were harvested at four hour intervals, the fresh weight measured, immediately frozen in liquid N_2_, ground to a fine powder and kept at −80 °C until analysis.

### Luciferase measurements

Arabidopsis was grown on media as described above, except that the plants were sown as clusters of five seedlings. Nine-day-old seedlings were dosed with 40 µl of 2 mM D-luciferin (nanolight) 1 h before dawn. Using a Berthold NightSHADE LB 985 *in vivo* Plant Imaging System, 10-day-old seedlings were imaged every hour for 800 sec following a 200 sec delay for chlorophyll fluorescence to decay. Photon counts per second were extracted using IndiGO software. Red and Blue LED light was provided at 80 µmol m^−2^ s^−1^. The change in photoperiod from LD to continuous light for 11-day-old seedlings was automated using IndiGO.

### Phase response curve

Arabidopsis was grown on custom assay boats^52^ and dosed as described above for luciferase measurements. Plants were transferred to constant low light (10 µmol m^−2^ s^−1^) at ZT0, and assay boats were transferred to liquid 0.5 MS + 90 mM sucrose for three hours to deliver a sugar pulse. Pulses were applied at intervals of 1.5 h during the first cycle in constant light. Following the sugar pulse assay, boats were briefly washed in liquid 0.5 MS to remove sugars and then returned to the assay plate. Luminescence imaging was automated every hour for four days as described above. The manual phase calculations and FFT-NLLS period estimations were performed using BRASS (amillar.org). Manual phase calculations were normalized to a 24 h period to give true phase (*x*):

6 $$x=\frac{\text{Manual phase calculation}}{\text{FFT}\mbox{-}\text{NLLS period estimation}}\times 24$$

### Statistical analysis of time-series starch data

Linear regression was applied in order to compare the rates of starch accumulation or loss between wild type (Col-0) and mutant (*prr7-11*) Arabidopsis. A set of starch time-series data was separated into the starch accumulation and the starch loss sections by the peak timing of starch concentration, and these two sections were analysed independently. For each section and genotype, linear regression was performed to assess starch level at dawn (intercept) and starch accumulation or decrease rate (slope). If the best-fit model estimated a negative intercept, we repeated the analyses by assuming that intercept is zero. We used analysis of covariance to test the statistical significance of the slope difference between Col-0 and *prr7-11*, which was represented as the synergetic effect of time × genotype. Detailed results of this analysis are presented in Supplementary Tables S2 and S3.

### Numerical analysis of mathematical models

A numerical analysis of the mathematical models was performed with *Mathematica* (version 10; Wolfram Research). Differential equations were solved numerically with the classical fourth-order Runge–Kutta method. Parameter values used for the analyses are listed in Supplementary Table S1.

## Electronic supplementary material

Supplementary Information

Supplementary Datasets 1–6
