# Supplementary material for: Adjustment of the Arabidopsis circadian oscillator by sugar signalling dictates the regulation of starch metabolism
Source: Sci Rep. 2017 Aug 16;7:8305. doi: 10.1038/s41598-017-08325-y (PMC5559614; doi:10.1038/s41598-017-08325-y)
Supplement: Supplementary file 1 — Supplementary Information [file 41598_2017_8325_MOESM1_ESM.doc]

**Supplementary Information:**

**Adjustment of the Arabidopsis circadian oscillator by sugar signalling dictates the regulation of starch metabolism**

Motohide Seki^1^, Takayuki Ohara^1,2^, Timothy J. Hearn^3^, Alexander Frank^3^, Viviane C. H. da Silva^4^, Camila Caldana^4,5^, Alex A. R. Webb^3^, Akiko Satake^1*^

^1^Department of Biology, Faculty of Science, Kyushu University, 744 Motooka, Nishi-ku, Fukuoka 819-0395 Japan

^2^Graduate School of Environmental Science, Hokkaido University, N10W5 Sapporo 060-0810 Japan

^3^Department of Plant Sciences, University of Cambridge, Cambridge CB2 3EA, United Kingdom

^4^Brazilian Bioethanol Science and Technology Laboratory (CTBE), Rua Giuseppe Máximo Scolfaro 10.000 CEP 13083-100 Campinas, São Paulo, Brazil

^5^Max Planck Partner Group at Brazilian Bioethanol Science and Technology Laboratory

^*^For correspondence: akiko.satake@kyudai.jp

**Supplementary Methods**

**An ideal function of the starch degradation rate for sucrose homeostasis and stationary sucrose levels**

We determined the nonlinear function for the starch degradation rate to formalize the linear starch profile and sucrose homeostasis. From Eqs. (2) and (4**)** in the main text, we can directly determine the functions for the starch degradation rate, *β*_L_,*_t_* and *β*_D_,*_t_*, for the light and dark periods, respectively.

First, by introducing *k*_L_ and *k*_D_ as the increased or decreased rate of starch level during the day and night, the right-hand side of Eq. (4**)** is simply rewritten as:

. (S1)

The constant rate of change in the starch level results in a linear profile for starch:

$C_{t}=\left\{\begin{matrix} k_{\text{L}}\{t\}+C_{\text{dawn}} \\ -k_{\text{D}}\left(\{t\}-\tau_{L}\right)+C_{\text{dusk}} \end{matrix}\right.\begin{matrix} \text{ } & \begin{matrix} \text{under light} \\ \text{under dark} \end{matrix} \end{matrix}$, (S2)

where *t* = 0 at a certain dawn, and {*t*} is a fractional part of *t* so that {*t*} = 0 holds at all dawns. Accordingly, {*t*} and {*t*} − *τ*_L_ represent the time after the most recent dawn and dusk, respectively. *C*_dawn_ and *C*_dusk_ in Eq. (S2) represent the level of starch at each dawn and dusk, respectively, and these parameters are assumed to be invariant from day to day under a given photoperiod with a constant light intensity.

For the light period, the starch degradation rate can be calculated with the following equation:

$\beta_{\text{L},t}=\frac{a\gamma-k_{\text{L}}}{\left(C_{\text{dawn}}+k_{\text{L}}\{t\}\right)^{\kappa}}=\frac{a\gamma-\frac{\left(C_{\text{dusk}}-C_{\text{dawn}}\right)}{\left(1-\tau_{\text{D}}\right)}}{\left[C_{\text{dawn}}+\frac{\left(C_{\text{dusk}}-C_{\text{dawn}}\right)\{t\}}{\left(1-\tau_{\text{D}}\right)}\right]^{\kappa}}$. (S3)

Similarly, for the dark period starch degradation rate can be calculated with the following equation:

$\beta_{\text{D},t}=\frac{k_{\text{D}}}{\left[C_{\text{dusk}}-k_{\text{D}}\left(\{t\}-1+\tau_{\text{D}}\right)\right]^{\kappa}}=\frac{\frac{\left(C_{\text{dusk}}-C_{\text{dawn}}\right)}{\tau_{\text{D}}}}{\left[C_{\text{dawn}}+\left(C_{\text{dusk}}-C_{\text{dawn}}\right)\left(\frac{1}{\tau_{\text{D}}}-\{t\}\right)\right]^{\kappa}}$. (S4)

Although Eqs. (S3) and (S4) include starch amount at dawn (*C*_dawn_) and dusk (*C*_dusk_), *C*_dusk_ is eliminated from these equations when sucrose homeostasis is considered [Eqs. (S17) and (S22)]. Therefore, our model is clearly different from the previous models because these models assuming that starch degradation rate is regulated by the amount of starch at dusk^14,15^. When plants do not sense the starch amount, as assumed in our model, an alternative mechanism to co-ordinate starch turnover under changing photoperiods is required. We hypothesize that this mechanism is the phase adjustment of the circadian oscillator by sucrose signals (Text S3). Using the light availability function *L_t_* with values of 1 for light and 0 for dark conditions, the starch degradation rate can be represented in general form:

$\beta_{t}=L_{t}\beta_{\text{L},t}+\left(1-L_{t}\right)\beta_{\text{D},t}$, (S5)

*β_t_* in Eq. (S5) is a non-linear and discontinuous function of time that has a peak at the end of dark period, namely at dawn (Fig. 2A). The discontinuous feature of *β_t_* at dusk implies that starch the starch degradation rate operates in different modes in the day and night due to dual regulation by the circadian oscillator and light signalling pathways^23^.

Perfect sucrose homeostasis is realized when sucrose is maintained at the same level regardless of light and dark conditions. To derive the condition for perfect sucrose homeostasis, we first determined the sucrose level for a stationary state under light and dark conditions. Because the starch level increases by Δ*C* = *C*_dusk_ − *C*_dawn_ during the light period, the rate of change in starch level under light is represented by the following equation:

$k_{\text{L}}=\frac{\Delta C}{\tau_{\text{L}}}=\frac{\Delta C}{1-\tau_{\text{D}}}$. (S6)

Using Eqs. (4), (S1), and (S6), we can derive the stationary level of sucrose under light conditions:

$\hat{S}_{\text{L}}=\frac{a-k_{\text{L}}}{H}=\frac{a-\frac{\Delta C}{1-\tau_{\text{D}}}}{H}$. (S7)

Similarly, we can derive the rate of change in the starch level under dark conditions:

$k_{\text{D}}=\frac{\Delta C}{\tau_{\text{D}}}$. (S8)

Using Eqs. (4), (S1), and (S8), the stationary level of sucrose during the dark period can be derived:

$\hat{S}_{\text{D}}=\frac{k_{\text{D}}}{H}=\frac{\frac{\Delta C}{\tau_{\text{D}}}}{H}$. (S9)

To realize perfect sucrose homeostasis, the difference between $\hat{S}_{\text{L}}$ and $\hat{S}_{\text{D}}$ must be zero. Based on Eqs. (S7) and (S9), this requirement can be formalized with the following equation:

. (S10)

From Eq. (S10), the necessary conditions for perfect sucrose homeostasis are defined with the following equation:

. (S11)

However, because starch degradation activity cannot be negative, the following inequality obtained from Eq. (S3) gives the upper bound for Δ*C*:

. (S12)

From Eqs. (S11) and (S12), perfect sucrose homeostasis is achieved only when *τ*_D_ ≤ *γ*, i.e., mild environments. If *τ*_D_ > *γ*, i.e., severe environments, is always larger than $\hat{S}_{\text{D}}$, and the difference in the sucrose level between light and dark periods is minimized when *β_t_* = 0 during the light period (Fig. 2B). Thus, we derived the stationary sucrose levels and starch degradation rates in two types of environments: mild and severe.

**Mild Environments (*τ*_D_ ≤ *γ*).** A mild environment means that the proportion of dark period in a day (*τ*_D_) is sufficiently small compared to the fraction of assimilated carbon allocated for starch production during the light period (*γ*). In a mild environment, plants can accumulate the starch required to keep the same sucrose level during the day and night. Substituting Eq. (S11) into Eqs. (S6)–(S9), we obtained the following equations:

$k_{\text{L}}=a\tau_{\text{D}}$, (S13)

$\hat{S}_{\text{L}}=\frac{a\tau_{\text{L}}}{H}$, (S14)

$k_{\text{D}}=a\tau_{\text{L}}$, (S15)

$\hat{S}_{\text{D}}=\frac{a\tau_{\text{L}}}{H}$. (S16)

Eqs. (S14) and (S16) specify the sucrose level under a perfect homeostasis state, $\hat{S}$, as *aτ*_L_/*H*. The starch degradation rate represented by Eq. (S5) can be further calculated:

$\beta_{t}=L_{t}\frac{a\left(\gamma-\tau_{\text{D}}\right)}{\left(C_{\text{dawn}}+a\tau_{\text{D}}\{t\}\right)^{\kappa}}+\left(1-L_{t}\right)\frac{a\tau_{\text{L}}}{\left[C_{\text{dawn}}+a\tau_{\text{L}}\left(1-\tau_{\text{D}}\{t\}\right)\right]^{\kappa}}$. (S17)

We can choose an arbitrary constant value for the parameter *C*_dawn_, and it is not necessary for the plant to sense starch level (*C_t_*) precisely for appropriate management of starch.

**Severe Environments (τ_D_ > γ).** A severe environment means that the proportion of dark period (*τ*_D_) is large compared to the fraction of assimilated carbon allocated for starch production (*γ*). The perfect sucrose homeostasis cannot be realized in this environment due to a shortage of carbon during the dark period. Substituting Δ*C* = *aγ*(1 − *τ*_D_) into Eqs. (S6)–(S9) results in the following equations:

$k_{\text{L}}=a\gamma$, (S18)

$\hat{S}_{\text{L}}=\frac{a\left(1-\gamma\right)}{H}$, (S19)

$k_{\text{D}}=\frac{a\gamma\left(1-\tau_{\text{D}}\right)}{\tau_{\text{D}}}$, (S20)

$\hat{S}_{\text{D}}=\frac{a\gamma\left(1-\tau_{\text{D}}\right)}{H\tau_{\text{D}}}$. (S21)

Subsequently, Eq. (S5) can be calculated as follows:

$\beta_{t}=\left(1-L_{t}\right)\frac{a\gamma\tau_{\text{D}}^{\kappa-1}\left(1-\tau_{\text{D}}\right)}{\left[C_{\text{dawn}}\tau_{\text{D}}+a\gamma\left(1-\tau_{\text{D}}\right)\left(1-\{t\}\right)\right]^{\kappa}}$. (S22)

Similarly to Eq. (S17), it is not necessary for the plant to sense the starch level (*C_t_*) precisely for appropriate management of starch. Equation (S22) indicates that starch degradation occurs only under dark conditions. The total amount of accumulated starch is not sufficient to keep the same sucrose level at night as the one observed during the day. This results in a lower sucrose level during the night when compared with the day.

Under the condition of *τ*_D_ ≤ *γ*, *k*_L_ increases with *τ*_D_ but *k*_D_ decreases with *τ*_D_. This theoretically-derived starch profile captures an important feature: the starch accumulation rate increases with a decreased photoperiod and starch degradation activity decreases with an increased photoperiod (Fig. 2C). When *τ*_D_ > *γ*, there is no increase in the starch accumulation rate during the decreased photoperiod because the starch degradation rate is zero (4, 6, and 8L in Fig. 2B). This result is consistent with the recent finding that starch accumulation rates are nearly the same under short-day conditions^22^.

**Independence of sucrose homeostasis from the threshold sucrose level**

Previous studies numerically determined the function of the starch degradation rate by minimizing the degree of sucrose deviation from a threshold sucrose level that is one of parameters of the model^16,17^. Here we determined that the starch degradation rate optimized to attain sucrose homeostasis is independent from the threshold sucrose level. In previous studies, it was assumed that there is a threshold sucrose level $\bar{S}$, and below or above this level causes stress to the plant^16,17^. Specifically, the cost function *P* is defined according to the following equation:

. (S23)

We set *t*_0_ = 0 and *t*_1_ = 1 so that Eq. (S23) represented the accumulated deviation from the favorable sucrose level $\bar{S}$ over one 24 h period (note that *τ*_L_ + *τ*_D_ = 1). We then examined all of the possible stationary carbon dynamics within this period. Here, we proved that minimization of the cost function *P* is independent of the value of the threshold sucrose level $\bar{S}$, which is approximately equivalent to minimizing $\Delta\hat{S}$ in Eq. (S10).

We started from the assumption that a plant maintains its sucrose level as $\hat{S}_{\text{L}}$ during day and $\hat{S}_{\text{D}}$ during night [Eqs. (S7) and (S9), respectively]. We then fixed the environmental parameters (*a*, *H*, *γ*, *τ*_L_, and *τ*_D_). $\hat{S}_{\text{L}}$ and $\hat{S}_{\text{D}}$ only depend on the free parameter Δ*C*, which is the difference between *C*_dawn_ and *C*_dusk_. Therefore, we have considered the situation that the carbon profile is governed solely by the parameter Δ*C*. The cost function *P*, which depends on two free parameters, Δ*C* and , can be calculated with the following equation:

. (S24)

Here, we assumed that the transition from $\hat{S}_{\text{L}}$ to $\hat{S}_{\text{D}}$ at dusk and the transition from $\hat{S}_{\text{D}}$ to $\hat{S}_{\text{L}}$ at dawn occur very fast, thus neglecting the time period needed for these transitions. The first and second terms in Eq. (S24) are independent of $\bar{S}$, whereas the third and the fourth terms depend on $\bar{S}$. Note that in the third term of Eq. (S24) represents the diel mean of the sucrose level.

We first showed that carbon profile (i.e., the value of Δ*C*) minimizing in Eq. (S24) is independent from the value of $\bar{S}$. Using Eqs. (S7) and (S9), we showed that in the third term of Eq. (S24), i.e., the diel mean of sucrose level, does not include the free parameter Δ*C*, regardless of whether the environment is mild or severe:

. (S25)

This indicates that the cost function *P* contains no cross terms of the two free parameters $\bar{S}$ and Δ*C*. Therefore, for any fixed value of $\bar{S}$, minimizing Eq. (S23) with respect to Δ*C* is the same as minimizing only the first and the second terms in Eq. (S23). Note that the right-hand side of Eq. (S24) represents the perfect sucrose homeostasis level in a mild environment [see Eqs. (S7) and (S9)] by definition of the temporal mean. In addition, we used Eq. (S23) to show that the value of $\bar{S}$ that minimizes *P* for any given value of Δ*C* is equal to , which represents the diel mean of sucrose level. Therefore, we are given the following equation:

. (S26)

From Eq. (S26), the cost function *P* becomes the diel variance of sucrose level, which can be zero only if the environment is mild (*τ*_D_ ≤ *γ*) and the perfect sucrose homeostasis is achieved.

Next, we show that minimizing *P* is equivalent to setting Δ*C* so as to minimize $\Delta\hat{S}$. The terms can be further calculated using Eqs. (S7) and (S9):

(S27)

The right-hand side of Eq. (S27) is a parabola function of Δ*C* minimized at Δ*C* = *aτ*_D_(1 − *τ*_D_), in which case $\Delta\hat{S}=0$ [Eqs. (S10) and (S11)]. Taking into account the fact that Δ*C* cannot exceed *aγ*(1 − *τ*_D_), we can conclude that *P* is minimized when Δ*C* = *aτ*_D_(1 −*τ*_D_) if *τ*_D_ ≤ *γ*, and when Δ*C* = *aτ*_D_(1 − *τ*_D_) otherwise (i.e., if *τ*_D_  > *γ*). In either case, $\Delta\hat{S}$ becomes the minimal value, as shown in the text.

**Light and sugar input functions**

We defined light and sugar input functions. We assumed that light pulses at dawn (satisfying {*t*} = 0) and dusk (satisfying {*t*} − *τ*_L_ = 0) set the phase of the oscillator so that it equals the external time; therefore, the light input term is defined with the following equation:

$\varepsilon_{\text{L}}Z_{\text{L}}(\varphi_{t})f_{\text{L}}(\tilde{L}_{t})=\left(t-\varphi_{t}\right)\delta(\{t\})+\left(t-\varphi_{t}\right)\delta(\{t\}-\tau_{\text{L}})$, (S28)

where *δ*(*x*) is Dirac delta function satisfying *δ*(0) = +∞ and *δ*(*x*) = 0 for *x* ≠ 0. The input from sugar signal is given as the Hill function of the sugar signal $\tilde{S}_{t}$:

(S29)

where *K* and *n* are constants, and

. (S30)

To define the sugar signal sensed by plants, $\tilde{S}_{t}$, we examined three different possibilities, as explained in the main text.

We determined the parameter values of *K* and *n* in Eq. (S29) in the text by minimizing the deviation from sucrose homeostasis under diverse photoperiod conditions. To this end, we first defined the fitness function with the following equation:

, (S31)

where $\bar{P}_{i}$ represents the cost defined in Eq. (S23) under a photoperiod of *τ*_L_ = *i*/24 when there is no phase shift. In contrast, *P_i_*(*K*, *n*) represents the cost under a photoperiod of *τ*_L_ = *i*/24 when there is phase shift. Because this cost is dependent on the response to sugar signals, it is a function of *K* and *n*. The fitness function measures the effect of phase shifts on sucrose homeostasis. When it is positive, a phase shift effectively reduces the deviation from sucrose homeostasis, otherwise it has no effect or a negative effect on sucrose homeostasis. The interval to evaluate $\bar{P}_{i}$ and *P_i_*(*K*, *n*) was set from the second day to the end of the fourth day after transfer from 10L:14D to other photoperiod conditions. We searched for parameters that maximized the fitness function.

**Two modes for starch degradation rates**

Equations (S3) and (S4) represent the starch degradation rate *β_t_* under light and dark conditions, respectively. To include the effect of phase shifts of the circadian clock on the starch degradation rate, we replaced the starch degradation rate *β_t_* in Eq. (S5) as $\tilde{\beta}(\phi_{t})$, which is a function of the phase at time *t* denoted as *ϕ_t_*. To define $\tilde{\beta}(\phi_{t})$, we considered two modes; the day mod [$\tilde{\beta}_{\text{L}}(\phi_{t})$] regulates the starch degradation rate during the day, while starch degradation rate at night is regulated by the night mode [].

Because the function of the day mode was defined as *β*_L_(*t*) in Eq. (S3**)** only during the subjective day, we needed to determine the function for the subjective night period. Here, we simply assumed that $\tilde{\beta}_{\text{L}}(\phi_{t})$ was given as a symmetrical function of *β*_L_(*t*) at the subjective dusk:

. (S32)

The function of the night mode was defined as *β*_D_(*t*) in Eq. (S4) only during the subjective night. Thus, we determined the function for the subjective light period. Similarly to $\tilde{\beta}_{\text{L}}(\phi_{t})$, we assumed that $\tilde{\beta}_{\text{D}}(\phi_{t})$ was given as a symmetrical function of *β*_D_(*t*) at the subjective dusk:

. (S33)

Using Eqs. (S32) and (S33), we derived the following equation:

. (S34)

**Numerical calculation of Phase Response Curve (PRC) for sucrose homeostasis**

We simulated physiological experiments by Haydon et al. (2013). In the experiment, a sugar pulse was added at different phases. We assumed the similar situation where sugar pulse Δ*S* was added and then computed the phase shift at each *t*_pulse_, denoted as *Z*_S_(*t*_pulse_), which minimizes the deviation from sucrose homeostasis defined as the following cost function:

, (S35)

where $\hat{S}$ stands for preferable sucrose level. As we showed in Text S2, the condition for sucrose homeostasis is independent from $\hat{S}$. *t*_0_ = 0 and *t*_1_ = 3/24 were used for our analysis, i.e. the cost function was evaluated over three hours after sucrose pulse was added. Transient changes of sucrose after sucrose pulse are described as:

. (S36)

The far-right term in Eq. (S36) means that a sucrose pulse Δ*S* is added at *t* = *t*_pulse_, where *δ*(*x*) is Dirac delta function satisfying *δ*(0) = +∞ and *δ*(*x*) = 0 for *x* ≠ 0. We chose Δ*S* = 10 for our numerical simulations. For each timing of sucrose pulse *t*_pulse_ = 0, 1/24, ..., 23/24 we determined the value of *Z*_S_ ∈ [−*τ*_D_, *τ*_L_] [Eq. (5)] that minimizes the cost function in Eq. (S35). As shown in Fig. 3B, phase responses that minimize the cost function in the simulations (the dots in Fig. 3B) were in almost complete accordance with the outcome of our thought experiment (the line in Fig. 3B).

**Predicting starch metabolisms under fluctuating whether conditions**

In addition to the seasonal change of photoperiod, we also examined the effect of short-term fluctuation in light intensity on the diel carbon profiles. When the light intensity was lowered to 50% of the original level [carbon capture rate *a* in Eq. (1) was reduced to half of the original value] and then returned to 100% the next day, the plant was predicted to accumulate more starch during the day; however, the plant rapidly reverted back to the original starch profile (Fig. S3A). The starch degradation rate was predicted to increase under the low light condition, but it decreased at almost zero level when the light intensity recovered (Fig. S3E). This flexible adjustment of starch metabolism did not occur if sugar entrainment was not considered (Fig. S3). These results suggest that phase shift in response to sugar signals is sufficient to control starch metabolism for different photoperiods and fluctuating weather conditions.

Table S1. Summary of parameters, variables, and functions.

|  | Definition | Units | Value |
| --- | --- | --- | --- |
| *a* | Carbon capture rate | μmolC_6_/gFW/day | 144 (Ref. 16) |
| *C_t_* | Starch concentration | μmolC_6_/gFW |  |
| *C*_dawn_ | Starch concentration at dawn | μmolC_6_/gFW | 0.15 |
| *C*_dusk_ | Starch concentration at dusk | μmolC_6_/gFW |  |
| *f*_L_(*L*) | Transformation function of light signal *L* |  |  |
| *f*_S_(*S*) | Transformation function of sucrose signal *S* |  |  |
| *H* | Sum of respiration rate and sucrose export rate | 1/gFW/day | 66.48 (Ref. 16) |
| *K* | Half saturation constant of *f*_S_(*S*) | μmolC_6_/gFW | 0.1 |
| *k*_D_ | Starch degradation rate under dark | μmolC_6_/gFW/day |  |
| *k*_L_ | Net starch accumulation rate under light | μmolC_6_/gFW/day |  |
| *L_t_* | Light condition (1 under light; 0 under dark) |  |  |
| *N* | Constant determining the shape of *f*_S_(*S*) |  | 1.0 |
| *S_t_* | Sucrose concentration | μmolC_6_/gFW |  |
| $\hat{S}_{\text{D}}$ | Sucrose concentration at homeostasis state under dark | μmolC_6_/gFW |  |
| $\hat{S}_{\text{L}}$ | Sucrose concentration at homeostasis state under light | μmolC_6_/gFW |  |
| *P* | Cost function that should be minimized | (μmolC_6_/gFW)^2^day |  |
| *t* | Time after the first dawn | Day |  |
| {*t*} | Time after the most recent dawn | Day |  |
| *t*_pulse_ | Time at which sugar pulse is added | Day |  |
| *Z*_L_(*ϕ*) | Continuous phase response curve to light signal |  |  |
| *Z*_S_(*ϕ*) | Continuous phase response curve to sugar signal |  |  |
| *β_t_* | Starch degradation activity | 1/gFW/day |  |
| *γ* | Carbon partitioning |  | 0.6 |
| Δ*C* | *C*_dusk_ minus *C*_dawn_ | μmolC_6_/gFW |  |
| *S* | $\hat{S}_{\text{L}}$ minus $\hat{S}_{\text{D}}$ | μmolC_6_/gFW |  |
| *δ*(*t*) | Dirac delta function |  |  |
| *κ* | Constant (starch degradation occurs in proportion to *C_t_^κ^*) |  | 2/3 |
| *τ*_D_ | Fraction of dark period in a day |  |  |
| *τ*_L_ | Fraction of light period in a day |  | 1 − *τ*_D_ |
| *ϕ* | Phase of the plant circadian clock | Day |  |
| *ω* | Angular frequency of the plant circadian clock |  | 1 |

**Table S2. Summary of linear regression analyses using starch data obtained from physiological experiments.**

|  |  |  | Level at dawn (µmol C_6_ g^−1^ FW) | | Accumulation/loss rate (µmol C_6_ g^−1^ FW h^−1^) | | |
| --- | --- | --- | --- | --- | --- | --- | --- |
| Transition | Photoperiod | Section | Col-0 | *prr7-11* | Col-0 | *prr7-11* | *p*-value |
| LD | 16L:8D | Accumulation | 4.51 ± 4.68 | (0) | 3.58 ± 0.47 | 7.77 ± 0.82 | < 0.001 |
| to |  | Loss | 0.83 ± 8.00 | (0) | 7.30 ± 1.45 | 7.76 ± 0.66 | 0.810 |
| SD | 8L:16D | Accumulation | (0) | (0) | 5.04 ± 0.56 | 4.54 ± 0.29 | 0.436 |
|  |  | Loss | 2.22 ± 3.47 | 8.75 ± 3.15 | 2.29 ± 0.34 | 1.89 ± 0.28 | 0.387 |
| SD | 8L:16D | Accumulation | 0.26 ± 3.37 | 0.19 ± 2.62 | 2.97 ± 0.65 | 3.86 ± 0.51 | 0.282 |
| to |  | Loss | (0) | (0) | 1.17 ± 0.17 | 1.64 ± 0.17 | 0.049 |
| LD | 16L:8D | Accumulation | 3.24 ± 3.50 | (0) | 3.28 ± 0.36 | 4.26 ± 0.23 | 0.028 |
|  |  | Loss | (0) | (0) | 7.03 ± 0.62 | 9.16 ± 0.60 | 0.018 |
| No | 12L:12D | Accumulation | 3.70 ± 2.73 | 1.85 ± 2.94 | 1.38 ± 0.32 | 3.90 ± 0.57 | < 0.001 |
| transition |  | Loss | 0.48 ± 1.83 | (0) | 1.64 ± 0.22 | 1.77 ± 0.11 | 0.613 |

**Note**: Estimated values ± standard errors are shown. Zero in parentheses means that the intersection (i.e., the starch level at dawn) was fixed to be zero. Accumulation rate values are slope of a linear regression of all the values of starch over the starch accumulation section; Loss rate values are slope of a linear regression of all the values of starch over the starch loss section. *p*-values in the rightmost columns indicate significance for differences of the accumulation or decrease rates between Col-0 and *prr7-11*.

**Table S3. Summary of linear regression analyses using the starch data obtained from the repeated experiment in Figure S9.**

|  |  |  | Level at dawn (µmol C_6_ g^−1^ FW) | | Accumulation/loss rate (µmol C_6_ g^−1^ FW h^−1^) | | |
| --- | --- | --- | --- | --- | --- | --- | --- |
| Transition | Photoperiod | Section | Col-0 | *prr7-11* | Col-0 | *prr7-11* | *p*-value |
| LD | 16L:8D | Accumulation | 3.25 ± 3.78 | 2.23 ± 4.87 | 2.53 ± 0.33 | 3.66 ± 0.48 | 0.058 |
| to |  | Loss | (0) | 1.14 ± 2.21 | 5.31 ± 0.28 | 5.28 ± 0.33 | 0.936 |
| SD | 8L:16D | Accumulation | 3.59 ± 4.56 | 7.82 ± 1.01 | 4.28 ± 0.88 | 4.79 ± 0.20 | 0.587 |
|  |  | Loss | (0) | (0) | 2.10 ± 0.17 | 2.65 ± 0.22 | 0.058 |
| SD | 8L:16D | Accumulation | 5.40 ± 4.09 | 5.68 ± 7.16 | 5.91 ± 0.77 | 5.14 ± 1.27 | 0.598 |
| to |  | Loss | 8.63 ± 4.16 | 8.51 ± 5.63 | 2.71 ± 0.41 | 2.56 ± 0.56 | 0.823 |
| LD | 16L:8D | Accumulation | (0) | (0) | 1.86 ± 0.19 | 3.15 ± 0.31 | < 0.001 |
|  |  | Loss | (0) | 3.10 ± 5.74 | 4.12 ± 0.67 | 6.45 ± 1.10 | 0.078 |

**Note**: Estimated values ± standard errors are shown. Zero in parentheses means that the intersection (i.e., the starch level at dawn) was fixed to be zero. Accumulation rate values are slope of a linear regression of all the values of starch over the starch accumulation section; Loss rate values are slope of a linear regression of all the values of starch over the starch loss section. *p*-values in the rightmost columns indicate significance for differences of the accumulation or decrease rates between Col-0 and *prr7-11*.


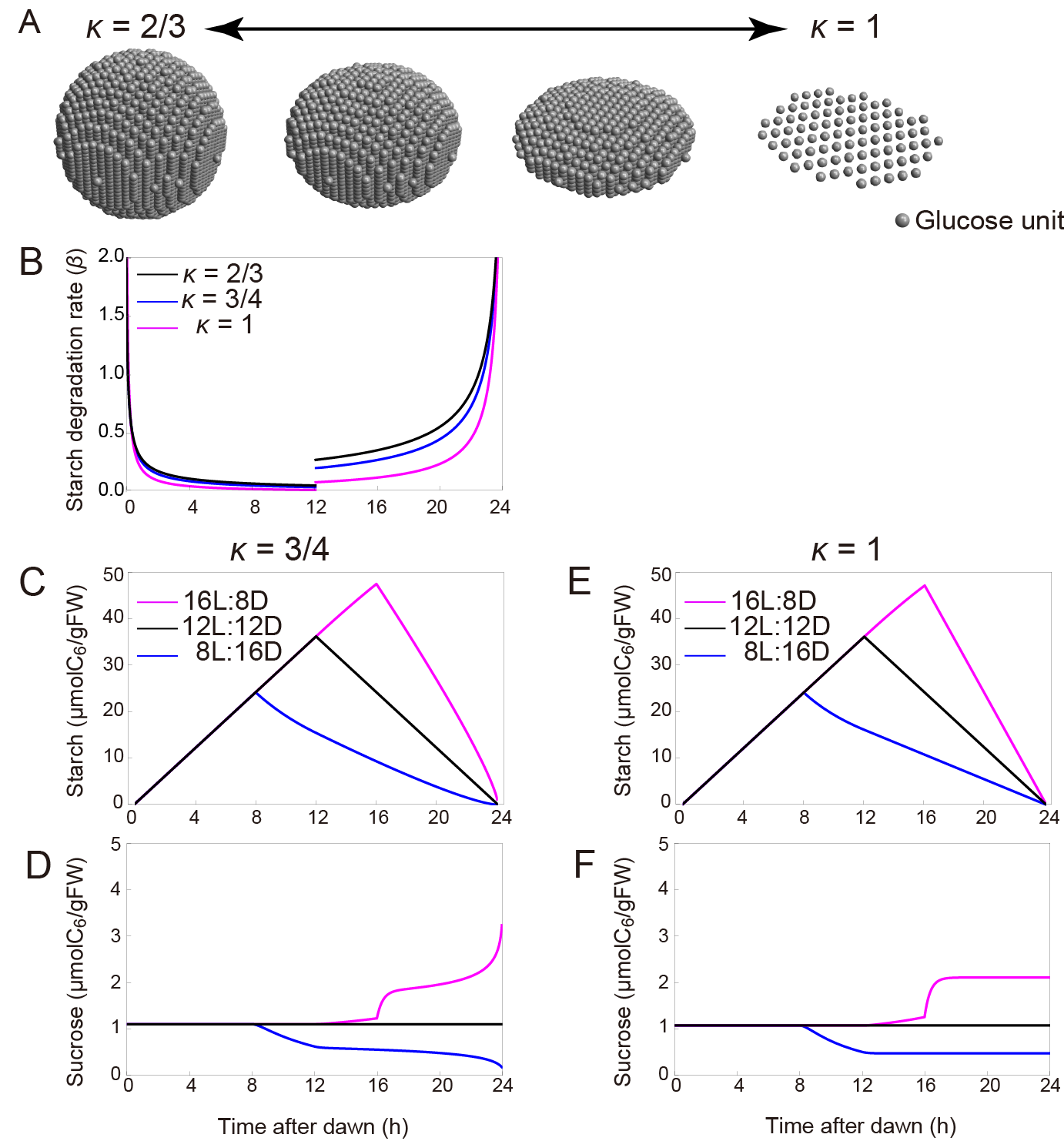


**Figure S1.** Effects of the allometry parameter (*κ*) regulating the shape of starch granule on the starch and sucrose profiles. (**A**) Different shapes of starch granules generated with the different value of *κ*. Small balls indicate glucose units in the polymer structure of starch granule. When *κ* = 2/3, the shape of starch granule is spherical, while it forms a carbon sheet when *κ* = 1. Arabidopsis starch granules in leaves are known to be discoid structures, suggesting that the value for *κ* lies between 2/3 and 1. (**B**) Ideal profiles for the diel starch degradation rate (*β*) for different values of *κ*. (**C**) Starch profiles and (**D**) sucrose profiles with *κ* = 3/4. (**E**) Starch profiles and (**F**) sucrose profiles with *κ* = 1. The model with different *κ *predicted the similar starch and sucrose profiles under the sudden change of photoperiod conditions from 12L:12D (black) to a long (16L:8D; purple) or short (8L:16D; blue) photoperiod. Parameter values used for the analyses are listed in Table S1.


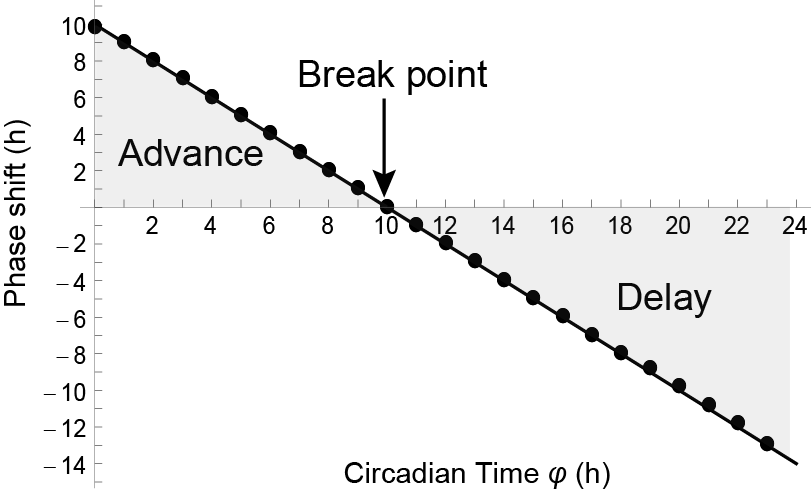


**Figure S2.** Optimal phase shift estimated from the simulated sugar-pulse experiment when the minimum starch degradation rate occurs at a circadian time of 10 h after subjective dawn. The line is the simulated experiment, and the dots were obtained from the numerical calculation (Text S5). The magnitude of the phase shift depends on the strength of the sugar stimulus and thus the magnitude of the phase shift is larger than the PRC presented in Fig. 3A because the maximum signal level was assumed for the simulated experiment.


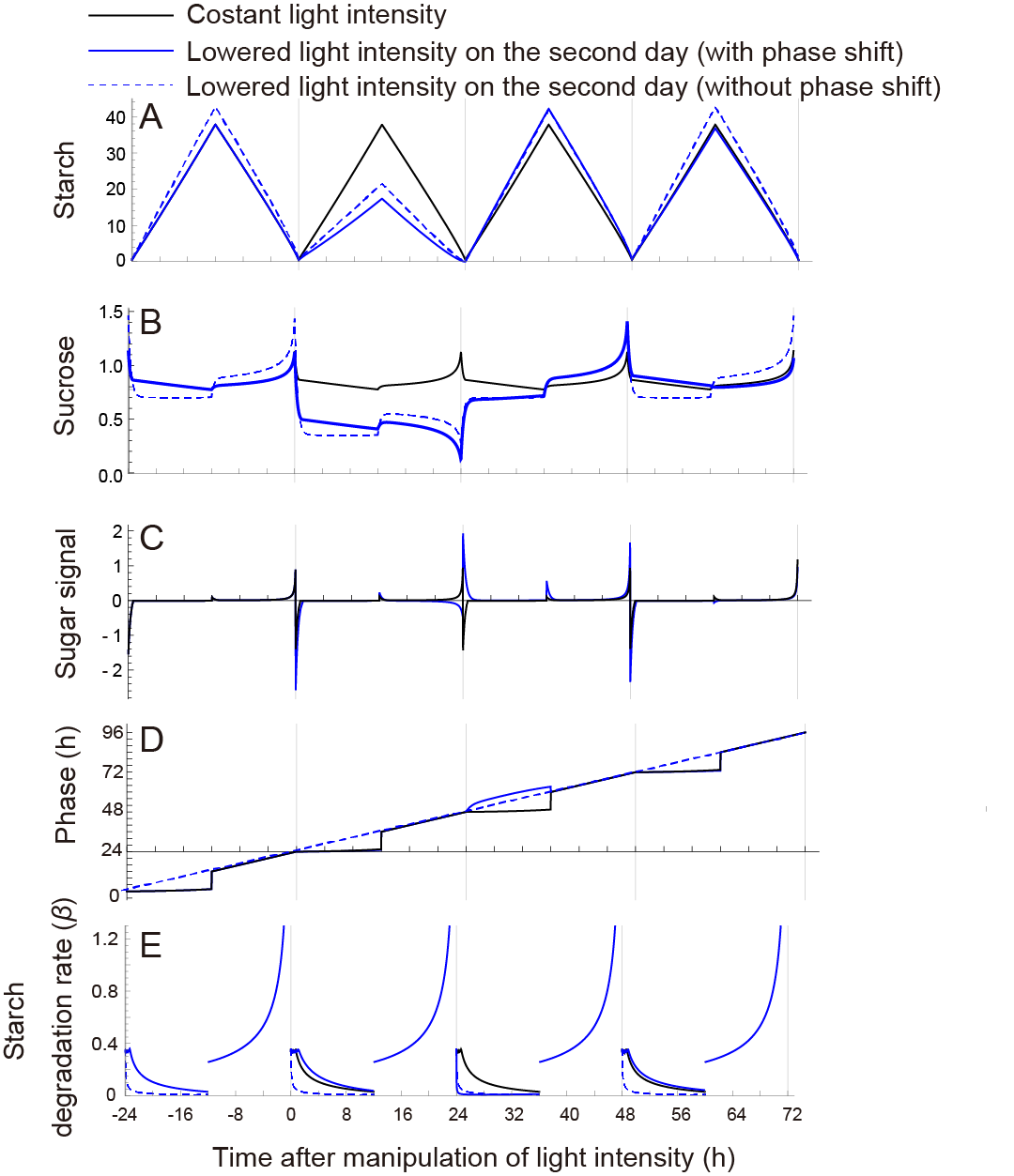


**Figure S3.** (**A**) Predicted starch profile, (**B**) sucrose profile, (**C**) sugar signal, (**D**) phase change, and (**E**) starch degradation rate of the plant that experienced half of the established light intensity on the second day. The unit for starch and sucrose is µmol C_6_ g^−1^ FW. The parameters used for the Hill function in Eq. (S29) are (*K*, *n*) = (0.1, 1.0).


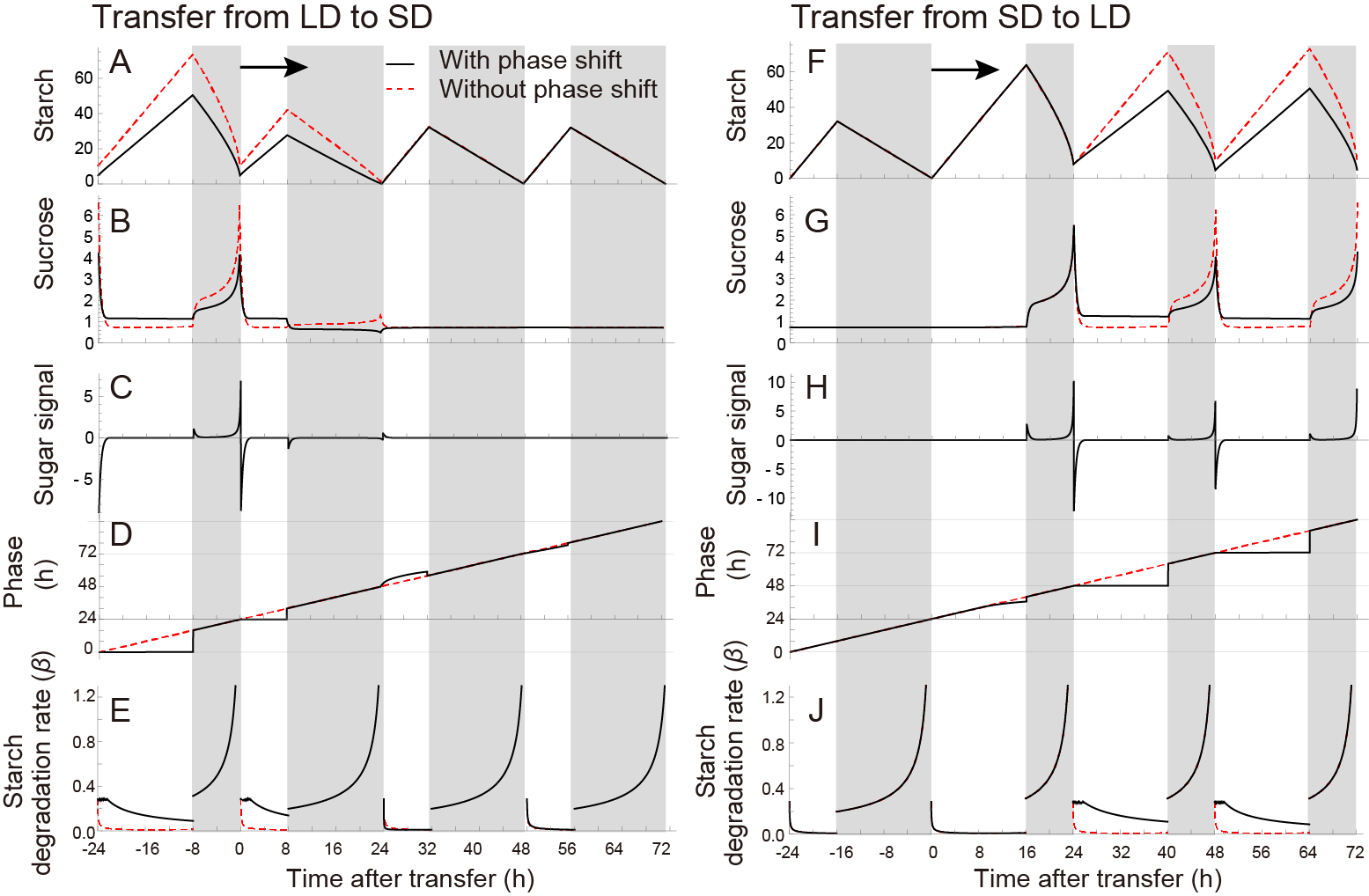


**Figure S4.** The predictions from the model when the break point of PRC is set at 8 h after subjective dawn by assuming that the subjective starch degradation rate is optimized for an 8 h photoperiod (i.e., $\hat{\tau}_{\text{L}}=8/24$). In these simulations we chose the parameter value *γ* = 0.68, with which the 8L:16D environment is a mild one for plants (Text S1). (**A**) Predicted starch profile, (**B**) sucrose profile, (**C**) sugar signal, (**D**) phase change, and (**E**) starch degradation rate of the plant that was transferred from long (16L:8D) to short (8L:16D) days. (**F**) Predicted starch profile, (**G**) sucrose profile, (**H**) sugar signal, (**I**) phase change, and (**J**) starch degradation rate of the plant that was transferred from short (8L:16D) to long (16L:8D) days. Black lines represent the plant with phase shift, and red lines represent the plant without phase shift. The unit for starch and sucrose is µmol C_6_ g^−1^ FW. The parameters used for the Hill function in Eq. (S29) are (*K*, *n*) = (0.1, 0.5).


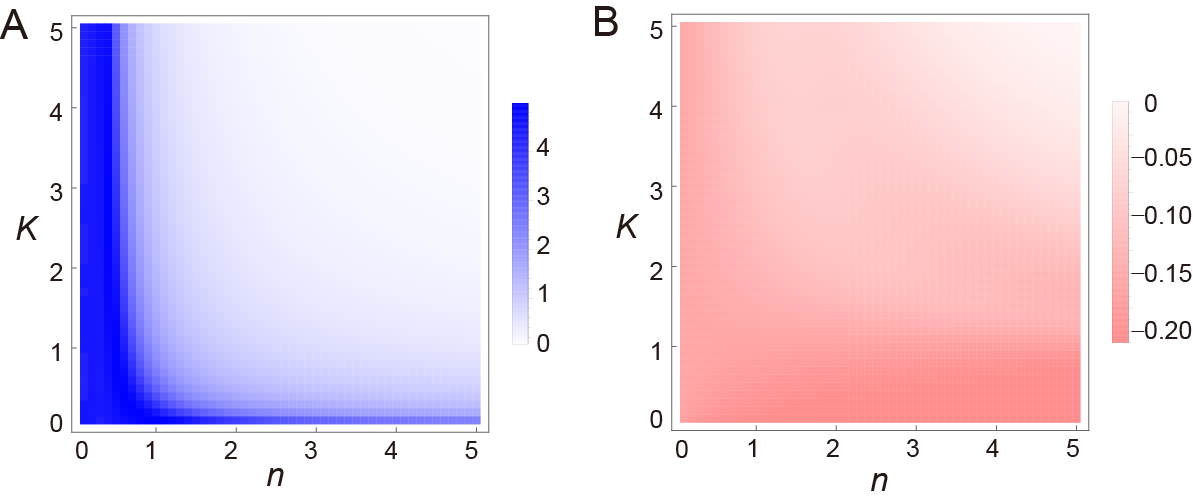


**Figure S5.** Density plot of scores indicating the degree of improvement for sucrose homeostasis by phase shift of the circadian oscillator. Diverse sets of parameter values (*K*, *n*) of the Hill function in Eq. (S29) were used to plot the score calculated by Eq. (S31). (**A**) The score generated by the model considering that plants sense the rate of change in sugar levels () and the sucrose concentration as a sugar signal (i.e., $\tilde{S}=S_{t}$). (**B**) The improvement of scores homeostasis (positive score) was realized only in (*A*). The break point of PRC was set as 10 h after subjective dawn.


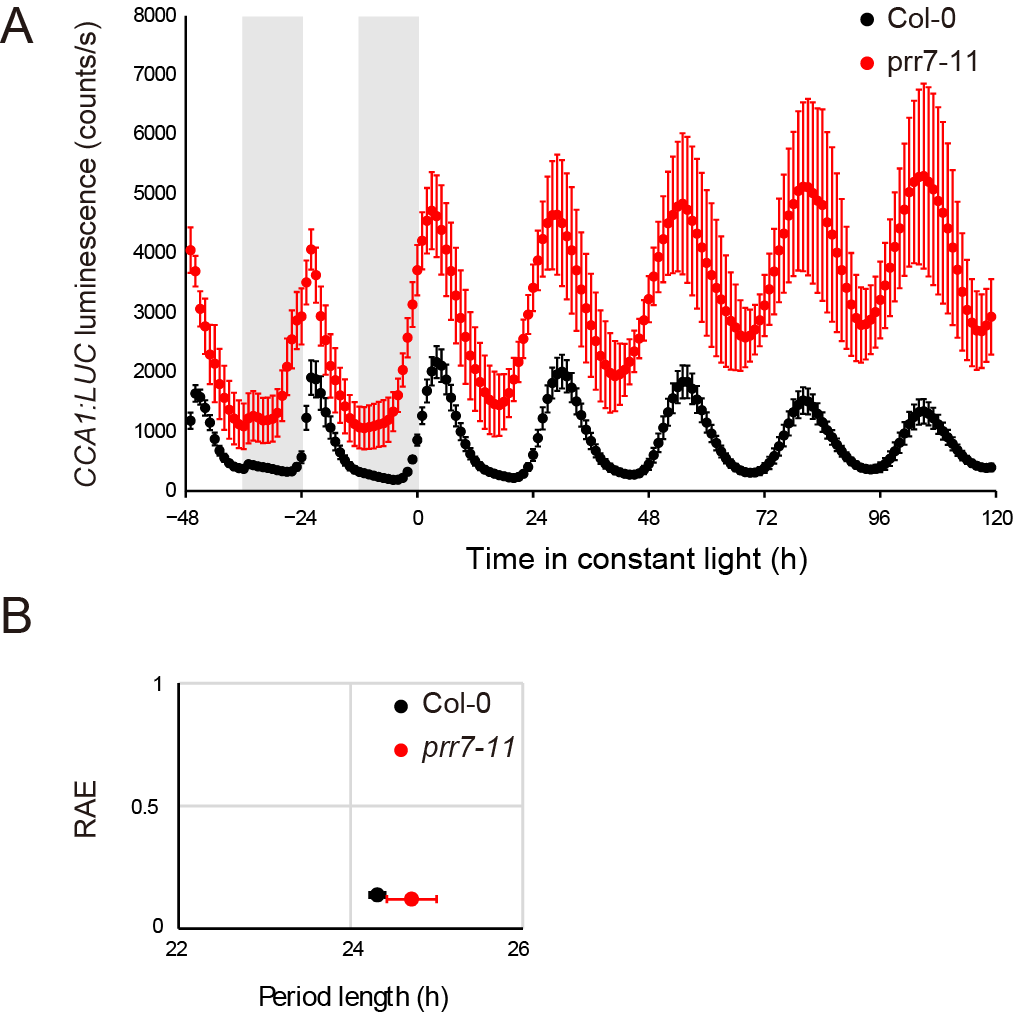


**Figure S6.** (**A**) *CCA1:LUC* luminescence for Col-0 (black) and *prr7-11* (red) in 80 µmol m^−2^ s^−1^ red/blue light measured for two light and dark cycles with a subsequent transfer to constant light for five days. The mean *CCA1:LUC* bioluminescence (counts s^−1^) is shown with the SEM. (**B**) The FFT-NLLS period estimate from *CCA1:LUC* data. The period and relative amplitude error (RAE) is shown with the SEM. Statistical analyses were conducted using biological replicates of *n* = 8.


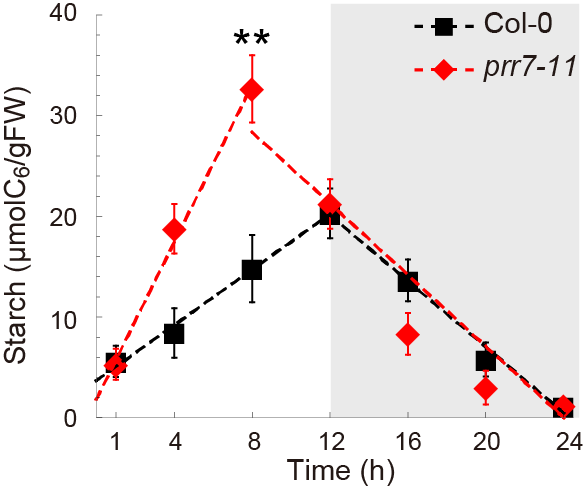


**Figure S7.** Plants were grown in 12:12 photoperiod and starch content calculated from rosettes of 21-day-old seedlings of Col-0 (black) and *prr7-11* (red). Mean starch (µmol C_6_ g^−1^ FW) ± SEM shown. Double asterisk (**) indicates *p* < 0.01 with Welch's *t*-test, *n* = 4–8. Dashed lines indicate linear regression lines for starch accumulation or loss (Supplementary Table S2). The experimental data are available online (Supplementary Dataset 4).


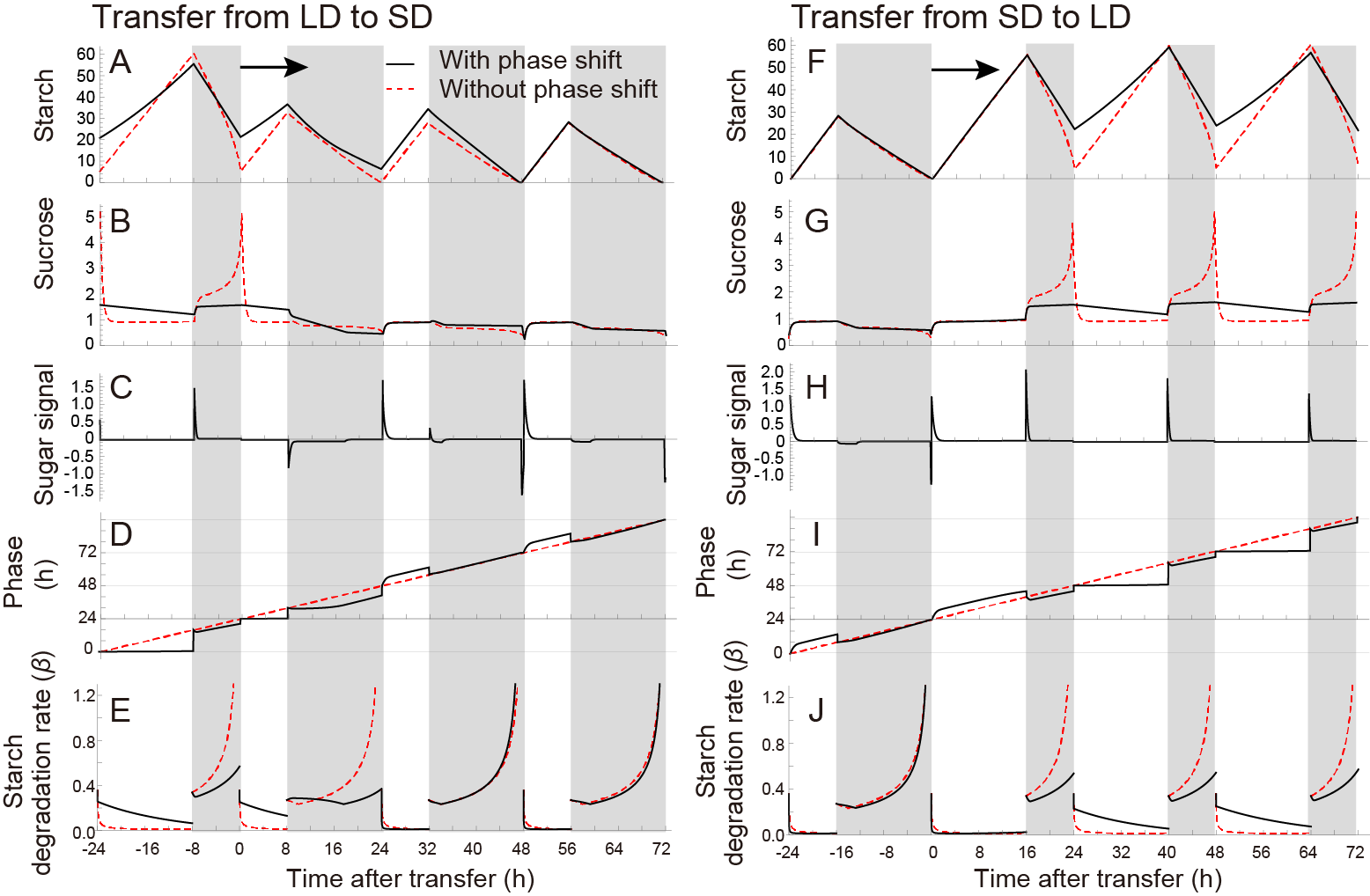


**Figure S8.** Predictions from the model considering the entrainment of the circadian clock by sugar during the day and night. (**A**) Predicted starch profile, (**B**) sucrose profile, (**C**) sugar signal, (**D**) phase change, and (**E**) starch degradation rate of the plant that was transferred from long (16L:8D) to short (8L:16D) days. (**F**) Predicted starch profile, (**G**) sucrose profile, (**H**) sugar signal, (**I**) phase change, and (**J**) starch degradation rate of the plant that was transferred from short (8L:16D) to long (16L:8D) days. Black lines represent the plant with phase shift, and red lines represent the plant without phase shift. The break point of PRC was set at 10 h after subjective dawn. The unit for starch and sucrose is µmol C_6_ g^−1^ FW. The parameters used for the Hill function in Eq. (S29) are (*K*, *n*) = (0.1, 1.5).


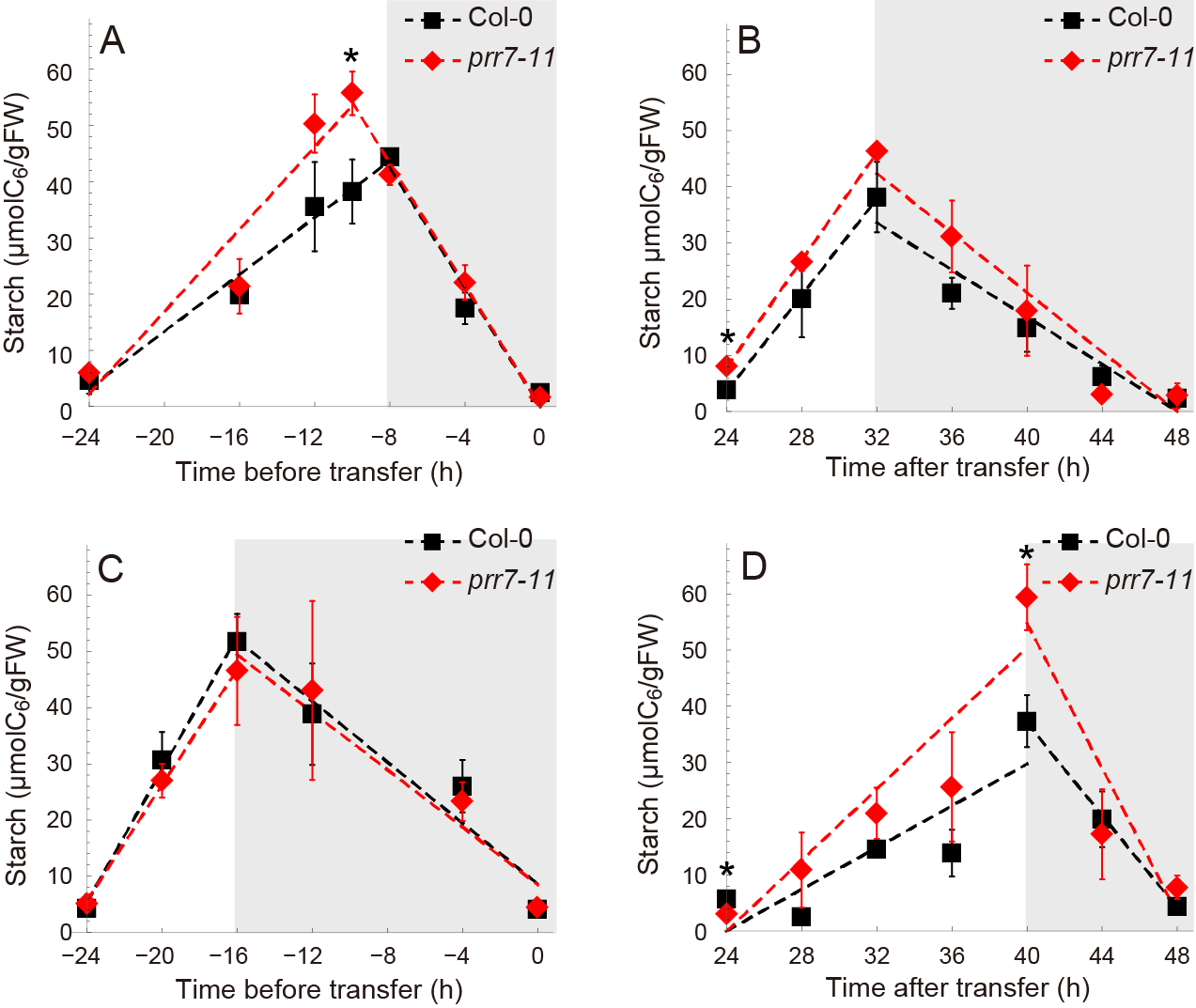


**Figure S9. Starch content in Col-0 and *prr7-11* plants grown in a 16L:8D photoperiod and harvested from 28-day-old seedlings independent of experiment in Figure 5.** (**A**) Col-0 and *prr7-11* plants grown in a 16L:8D photoperiod and harvested from 28-day-old seedlings and (**B**) 30-day-old seedlings that were transferred to 8L:16D after growing in standard LD conditions for 28 days. (**C**) The starch content in Col-0 and *prr7-11* grown in 8L:16D photoperiod and harvested from 28-day-old seedlings and (**D**) 30-day-old seedlings that were transferred to 16L:8D after growing in SD for 28 days. The light intensity was 100 µmol m^−2^ s^−1^. Asterisk (*) indicates *p* < 0.05 with Welch's *t*-test, A–B *n* = 3, C–D *n* = 3 or 5. Dashed lines indicate linear regression lines for starch accumulation or loss (Table S3). The experimental data are available online (Supplementary Datasets 5–6).
